# Alpha Carbonic Anhydrase 5 Mediates Stimulation of ATP Synthesis by Bicarbonate in Isolated *Arabidopsis* Thylakoids

**DOI:** 10.3389/fpls.2021.662082

**Published:** 2021-08-26

**Authors:** Tatiana P. Fedorchuk, Inga A. Kireeva, Vera K. Opanasenko, Vasily V. Terentyev, Natalia N. Rudenko, Maria M. Borisova-Mubarakshina, Boris N. Ivanov

**Affiliations:** ^1^Institute of Basic Biological Problems, Federal Research Center “Pushchino Scientific Center for Biological Research of the Russian Academy of Sciences”, Pushchino, Russia; ^2^Centre for the Analysis of Genome Evolution and Function (CAGEF), University of Toronto, Toronto, ON, Canada

**Keywords:** thylakoid membrane, ATP synthase, bicarbonate, carbonic anhydrase, photophosphorylation

## Abstract

We studied bicarbonate-induced stimulation of photophosphorylation in thylakoids isolated from leaves of *Arabidopsis thaliana* plants. This stimulation was not observed in thylakoids of wild-type in the presence of mafenide, a soluble carbonic anhydrase inhibitor, and was absent in thylakoids of two mutant lines lacking the gene encoding alpha carbonic anhydrase 5 (αCA5). Using mass spectrometry, we revealed the presence of αCA5 in stromal thylakoid membranes of wild-type plants. A possible mechanism of the photophosphorylation stimulation by bicarbonate that involves αCA5 is proposed.

## Introduction

ATP production in chloroplasts under illumination [photophosphorylation (PP)] is required for the key step of photosynthesis—the inclusion of CO_2_ into organic compounds in the Calvin–Benson cycle, which proceeds in the chloroplast stroma. PP is accomplished in chloroplast thylakoid membranes (Thyl) at the expense of proton motive force (*pmf*), which is established across these membranes as a result of the photosynthetic electron transfer occurring in the light. In thylakoid membranes, *pmf* is represented mostly as a difference between pH values inside and outside thylakoids, ΔpH. ATP synthesis is catalyzed by the chloroplast coupling factor, CF1, the part of ATP-synthase complex exposed to the chloroplast stroma. In the early 1960s, the stimulation of PP in isolated thylakoids by adding bicarbonate (HCO_3_^–^) to thylakoid suspension was discovered (Punnett and Iyer, 1964). Later findings showed that HCO_3_^–^ also stimulates other types of ΔpH-dependent ATP synthesis in thylakoids, namely, ADP phosphorylation after turning off actinic light and phosphorylation initiated by acid–base transition in the dark (Cohen and Jagendorf, 1972). The PP rate increases along with the increase in bicarbonate concentration (Harris, 1978).

The observed effect of bicarbonate was proposed to be related to its influence on the interaction between energized thylakoid membranes and CF1 (Cohen and Jagendorf, 1972). The bicarbonate effect was found to be more pronounced in pH range of 7.0–7.4, where PP rate was far from its maximum rate. It was suggested that the stimulatory effect of bicarbonate on ATP synthesis could be attributed to its ability to affect the conformation of CF1 directly (Cohen and MacPeek, 1980). However, CF1 conformation changes, which can be measured as a change in the Mg^2+^-ATPase activity of purified CF1 (Nelson et al., 1972), were shown to be activated by addition of anions other than bicarbonate such as carbonate, borate, and sulfite (Malyan, 2003). At the same time, the stimulatory effect on PP was not observed in experiments with anions of weak acids, such as acetate, fumarate, and sulfite (Fedorchuk et al., 2018). The latter data were in accordance with the results by Avdeef et al. (1982), who found that anions such as azide, chloride, and nitrate, unlike bicarbonate, had no stimulatory effect on PP in chromatophores isolated from photoautotrophic bacteria *Chromatium* sp.

Podorvanov et al. (2005) and Onoiko et al. (2010) have suggested that carbonic anhydrase (CA), the enzyme catalyzing both the hydration of carbon dioxide (CO_2_) to protons and HCO_3_^–^, and dehydration of bicarbonate leading to production of CO_2_ and water, could engage in bicarbonate stimulation of PP. The authors proposed this statement based on suppression of this stimulation in the presence of CA inhibitors, such as acetazolamide or ethoxzolamide. However, these inhibitors suppress electron transfer directly since they interact with the components of the photosynthetic electron transfer chain (PETC) (Swader and Jacobson, 1972; Graham et al., 1974; Fedorchuk et al., 2018). Such inhibition of electron transfer would lead to decrease of *pmf* and consequently to PP suppression.

In our previous study, we investigated the effect of a hydrophilic CA inhibitor mafenide on the stimulation of PP by bicarbonate, using thylakoids isolated from pea leaves (Fedorchuk et al., 2018). At the mafenide concentrations low enough to have no negative effect on the electron transfer rate and on the rate of PP in the absence of HCO_3_^–^, it significantly decreased the stimulation of PP in the presence of HCO_3_^–^. This suggested the involvement of CA in this stimulation since HCO_3_^–^ is one of the substrates of CA.

A number of studies demonstrated that there are several CAs present in thylakoids of higher plants (Lu and Stemler, 2002; Pronina et al., 2002; Ignatova et al., 2006; Rudenko et al., 2007). The soluble CA was discovered in the thylakoid lumen (Rudenko et al., 2007; Fedorchuk et al., 2014). At least two CAs were found in the granal thylakoid membranes close to photosystem II (PSII), and one CA was found in the stromal thylakoid membranes (STM) (Ignatova et al., 2006, 2011; Rudenko et al., 2006), which contain mainly photosystem I (PSI) complexes and ATP-synthase complexes.

In the present study, we scrutinized the stimulation of PP by bicarbonate in *Arabidopsis* thylakoids. The genome of this plant is fully sequenced, allowing us to study the nature of this phenomenon using mutants with knocked-out genes of enzymes of interest. We have shown that PP is stimulated by bicarbonate in thylakoids isolated from leaves of *Arabidopsis thaliana* wild-type (WT) plants and that this stimulation was absent in thylakoids from the mutant lacking CA αCA5 (according to the nomenclature proposed by Fabre et al., 2007). Mass spectrometry analysis allowed us to prove the location of αCA5 in STM. The mechanism of PP stimulation by bicarbonate and possible role of this stimulation *in vivo* is discussed.

## Materials and Methods

### Plant Material

*Arabidopsis thaliana* (L.) ecotype Columbia (WT) plants and *Arabidopsis* plants with knocked-out *At1g08065* gene, encoding αCA5 (αCA5-KO), were grown in a growing chamber at 22/19°C, illumination of 100 μmol quanta m^–2^ s^–1^ and day length of 8 h at ambient CO_2_ concentration. The seeds of the mutants were obtained from the *Arabidopsis* Biological Resource Center as T-DNA insertion lines (SALK_097331C and SALK_038466C), and the corresponding homozygous mutant plants were used (“9–2” and “9–14” lines, respectively) in the present study (Supplementary Figure 1). The mutants differed in the positions of the gene knockout insertion. The positions of T-DNA insertions in two lines of αCA5-KO are shown in Supplementary Figure 2.

### Quantitative Reverse Transcription PCR and Agarose DNA Electrophoresis

RNA was isolated using the Aurum total RNA Mini Kit (Bio-Rad) from leaves of WT plants and αCA5-KO, “9–2” and “9–14” lines, previously frozen in liquid nitrogen and treated with DNase to eliminate any genomic DNA contamination. Reverse transcription was performed using the reverse transcription kit OT-1 (Sintol) with oligo (dT) as a primer. The resulting cDNAs and specific primer pair to *At1g08065* gene (forward 5′-TCTCCTCACGTTGGAAAGATACTTGAAG-3′ and reverse 5′-TTGTTTTAATGTCACAGTCCTCATCTC-3′) were designed to span exon–exon junctions and used for the first step of PCR with predicted PCR product of 202 base pair (bp). The quantitative reverse transcription PCR (qRT-PCR) was performed using qPCRmix-HS SYBR (Evrogen) in LightCycler 96 Instrument, Roche Diagnostics GmbH. The content of the PCR product was insufficient to obtain a fluorescence signal, and PCR product was used as DNA template for the second PCR step using the “internal” primers, i.e., primers designed complementary to the PCR product obtained in the first step of the PCR (forward 5′-AAGAGGATAACTGATACACACGAATC-3 and reverse 5′-ATCGTCCAAATCACATTTTCAGAAC-3′), with predicted PCR product of 143 bp. QRT-PCR was performed as described above with housekeeping ubiquitin-encoding gene (forward 5′-TGCTTGGAGTCCTGCTTGGA-3′ and reverse 5′-TGTGCCATTGAATTGAACCCTCT-3′) as a control. The ubiquitin-encoding gene was expressed in WT and αCA5-KO of both lines, whereas α*ca5* (*At1g08065*) gene expression was observed only in WT (Supplementary Figure 3A). The resulting PCR products were used for electrophoresis in 1% agarose gel with 40 mM of Tris acetate buffer in the presence of 1 mM of EDTA, and ethidium bromide was used as an intercalating agent. The DNA ladder with DNA fragments ranging from 100 to 3,000 bp (SibEnzyme, Russia) was used as DNA size markers. *At1g08065* gene transcripts were absent in αCA5-KO and were present in WT plants with PCR product size corresponding to the predicted one (Supplementary Figure 3B).

### Isolation of Thylakoids and Stromal Thylakoid Membranes

Thylakoids were isolated from leaves of 1.5 to 2-month-old *Arabidopsis* plants, according to Ignatova et al. (2011), with modifications. Bovine serum albumin (BSA) at concentration of 1% was added to all media (Fedorchuk et al., 2014). To isolate STM, thylakoids were pushed through a French Press (Thermo Electron, United States) with the pressure of 1,000 psi. Then thylakoid membranes were incubated for 30 min with stirring on the ice bath with Triton X-100 at a Triton/chlorophyll (Chl) ratio (w/w) of 0.7 followed by centrifugation at 12,000 × g for 20 min. The pellet was resuspended in the medium containing 0.4 M of sucrose, 50 mM of Mes-KOH (pH 6.5), 5 mM of MgCl_2_, 35 mM of NaCl, 20 mM of sodium ascorbate, 10 mM of HCO_3_^–^, 2 mM of EDTA-Na, 5 mM of phenylmethylsulfonyl fluoride, 1 mM of α-aminocaproic acid, and 1 mM of benzamidine and incubated under stirring for 30 min on ice with *N*-dodecyl-β-D-maltoside (DM) at DM/Chl ratio of 3.4. Thylakoid membranes were precipitated by centrifugation at 32,000 × g for 40 min. The supernatant was doubly diluted with cold distilled water, and STM was precipitated by centrifugation at 80,000 × g for 1 h. All procedures were carried out at 4°C. The obtained membranes were frozen in liquid nitrogen after addition of glycerol to 20% and stored at –80°C.

### Chlorophyll Content Measurements

The Chl content was determined in ethanol extracts according to Lichtenthaler (1987).

### The Chlorophyll *a* Fluorescence Spectrum Measurements

Low-temperature Chl *a* fluorescence spectra at 77K were measured according to Cederstrand and Govindjee (1966) using spectrofluorometer (Hitachi, Japan) and applying a monochromatic exciting light with wavelength of 435 nm.

### Denaturing Electrophoresis and Western Blot Analysis

Denaturing electrophoresis was performed according to Schägger and von Jagow (1987) in 15% polyacrylamide gel (PAAG) in Mini-PROTEAN Cell (BioRad). Samples of thylakoid membranes were diluted in the loading buffer (pH 6.8), containing 60 mM of Tris-HCl, 2% sodium dodecyl sulfate, 10% sucrose, 0.05% bromophenol blue, and 5% dithiothreitol, heated at 99°C for 2 min. Insolubilized material was precipitated by centrifugation at 10,000 rpm in Centrifuge MiniSpin (Eppendorf) for 10 min. Samples of denatured proteins from Thyl and STM corresponding to 3 μg of Chl content were loaded on gel. Prestained standard kit in dual color (10–250 kDa) (Bio-Rad, United States) was used as the protein molecular mass markers.

After electrophoresis, proteins were transferred onto polyvinylidene difluoride (PVDF) membrane (BioRad, United States) using wet blotting system Mini Trans-Blot Cell (BioRad, United States). Western blot analysis was performed according to Onda et al. (2000) with anti-rabbit primary antibodies against PsbA and PsaC (Agrisera) (AS05 084 and AS10 939, correspondingly). Goat anti-rabbit antibodies labeled with alkaline phosphatase (Agrisera) were used as secondary antibodies in dilution of 1:5,000. The antibody–antigen conjugates were detected by Alkaline Phosphatase Conjugate Substrate Kit (BioRad, United States).

### Isolation of Carbonic Anhydrase From Stromal Thylakoid Membranes

The preparations of STM were incubated with stirring on an ice bath with Triton X-100 at a Triton/Chl ratio (w/w) of 35.0. After slow addition of pre-cooled acetone (0°C) to acetone/Chl ratio (v/v) of 7/10, they were incubated with stirring on an ice bath for 10 min with subsequent centrifugation at 12,000 × g for 10 min (Scopes, 1987). Precipitate containing membrane proteins was solubilized in the buffer containing 6 M of urea, 50 mM Tris-HCl (pH 8.0), 1 M of NaCl, 0.1% DM, and 0.1% Triton X-100 (Buffer 1). Affinity chromatography was carried out by loading proteins onto a column filled with agarose/mafenide (Sigma, United States). After incubation for 40 min, the column was washed with Buffer 1 to remove non-specifically bound substances. CA was eluted from the column with Buffer 1 containing 50 μM of mafenide, which was then washed out by centrifugation of the eluate in Millipore concentrators to restore CA activity.

### Non-denaturing Electrophoresis

Non-denaturing electrophoresis was performed according to Peter and Thornber (1991), with modifications. Before loading on gel, DM was added to purified protein sample at a protein/DM ratio (w/w) of 5. The electrophoresis was carried out in 10% PAAG at a current of 3–5 mA overnight at 4°C in darkness. Coomassie Brilliant Blue G-250 staining was applied for protein visualization. The activity of CA in PAAG was visualized after incubation of the gel on ice for 20–30 min in 44 mM of veronal buffer (pH 8.1) with bromothymol blue followed by transfer into water saturated with CO_2_ at 0°C (Edwards and Patton, 1966). Blue gel turned yellow where CA activity was present.

### Mass Spectrometry Analysis

After Coomassie staining, a piece of gel with protein band that contained the protein with the CA activity was incubated with 50 mM of ammonium bicarbonate (pH 7.8), and then the released proteins were subjected to reduction with dithiothreitol at 56°C, alkylation with iodoacetamide at room temperature, and overnight digestion with sequencing-grade trypsin (Promega, Madison, WI) at 37°C. The enzymatic reactions were stopped with 3% formic acid, and peptides were purified and concentrated with Pierce C18 Spin Columns (Thermo Fisher Scientific) and dried to a pellet under vacuum. Peptide samples were then solubilized in 0.1% formic acid prior to liquid chromatography–tandem mass spectrometry (LC-MS/MS) analyses. Subsequent analytical separation for LC-MS/MS analysis of proteins, chromatography, and mass spectrometry was performed according to Lee et al. (2018).

Samples were separated on an EASY-nLC 1,200 nano-LC system (Thermo Fisher Scientific), injected into LTQ Orbitrap XL^TM^ mass spectrometer (Thermo Fisher Scientific, United States) through the nano spray source (Proxeon, Odense, Denmark). Spectrum and peak list generation was performed using Xcalibur 2.2 (Thermo Fisher Scientific, United States).

Proteins were identified by searching all MS/MS spectra against a large database composed of the complete proteome of *A. thaliana* ecotype Columbia (Taxon identifier 3702; UniProt proteome ID UP000006548) using SEQUEST (Thermo Scientific Proteome Discoverer software).

### Measurements of Photophosphorylation and Electron Transfer Rates

For measurements of ATP synthesis and photosynthetic electron transfer rate, thylakoids were isolated from leaves of 3 to 4-weeks-old *Arabidopsis* plants of WT or mutant as described in Casazza et al. (2001) and resuspended in buffer containing 0.3 M of sorbitol, 2.5 mM of EDTA, 5 mM of MgCl_2_, 10 mM of NaHCO_3_, 20 mM of HEPES (pH 7.6), and 0.5% BSA and stored on ice. Experiments were performed on the same day as isolation. Plants were kept in the light for 2–3 h prior to isolation of thylakoids.

The light-induced rate of photosynthetic electron transfer with methyl viologen (MV), the artificial electron acceptor, was measured as the rate of oxygen consumption in a temperature-controlled glass cell at 21°C, using Clark-type pO_2_-electrode. Illumination of 500 μmol quanta m^–2^ s^–1^ was provided with a light-emitting diode (Epistar, 660 nm). The reaction medium contained 0.1 M of sucrose, 20 mM of NaCl, 5 mM of MgCl_2_, 20 μM of MV, 50 mM of HEPES-KOH (pH 7.6), and thylakoids with 20 μg Chl ml^–1^. According to protocol, 4 mM of NaHCO_3_, 0.2 mM of ADP, 2 mM of NaH_2_PO_4_, and 1 μM of gramicidin D were added, where indicated.

PP rate was measured in the reaction mixture containing 0.2 mM of ADP and 2 mM of K_2_HPO_4_, 2 mM of HEPES-KOH (pH 7.5), 10 mM of NaCl, 5 mM of MgCl_2_, 0.1 M of sucrose, and 50 μM of phenazine methosulfate (PMS) or 20 μM of MV, as the rate of pH increases under illumination of thylakoids with white light (400 μmol quanta m^–2^ s^–1^) at 22°C for 1–2 min. The amount of absorbed protons was determined by titration of the medium with HCl, and the rate of ATP synthesis was calculated according to Nishimura et al. (1962). 2 min before the rates of both ATP synthesis and electron transfer were measured, 4 mM of HCO_3_^–^, 4 mM of NH_4_Cl, and mafenide at indicated concentrations were added.

## Results

### Effects of Mafenide, the Carbonic Anhydrase Inhibitor, on Electron Transfer Rate and on the Stimulation of Photophosphorylation by Bicarbonate in *Arabidopsis* Thylakoids

To elucidate the role of CA in the stimulation of PP by bicarbonate, the effect of CA activity inhibition was studied. Taking into account the possible inhibitory effect of some CA inhibitors on the rate of electron transfer (Swader and Jacobson, 1972; Fedorchuk et al., 2018), the effect of mafenide, a hydrophilic inhibitor of CAs, on this rate was assessed. The measurements of the light-induced photosynthetic electron transfer rate in isolated thylakoids were carried out in the presence of MV, the effective electron acceptor at PSI. In the thylakoids from WT plants, the addition of ADP and inorganic phosphate to the thylakoid suspension noticeably increased the electron transfer rate (transfer coupled with ATP synthesis) as compared with the rate in the presence of MV only (basal transport) (Table 1). The rate was even higher in the presence of gramicidin D as an uncoupler (uncoupled transport) (Table 1). It means that the rate of electron transfer was limited by intrathylakoid (lumen) pH, which decreases in the light under basal conditions, but increases under coupling and uncoupling conditions when there is proton efflux from the lumen through either ATP-synthase proton channels or gramicidin pores, correspondingly. These data indicate that isolated thylakoid membranes were rather tightly coupled.

**TABLE 1:** Effect of mafenide and bicarbonate on electron transfer rates in *Arabidopsis thaliana* thylakoids.

Electron transfer type	The electron transfer rate, μmol O_2_/mg Chl × h				
	
	Mafenide				4 mM of HCO_3_^–^
	
	–	0.1 mM	0.25 mM	0.5 mM	
Basal	27.2 ± 2.3^A^	26.9 ± 5.1^A^	n.d.	n.d.	26.8 ± 4.8^A^
Coupled	49.3 ± 3.1^A^	51.8 ± 8.2^A^	41.8 ± 2.1^B^	30.6 ± 2.5^C^	37.5 ± 3.2^D^
Uncoupled	84.1 ± 8.0^A^	77.4 ± 3.2^A^	63.4 ± 2.9^B^	54.36 ± 2.9^C^	79.8 ± 6.4^A^

*The basal transfer rate was measured in the presence of 20 μM of MV as an acceptor; the coupled transfer rate was measured in the presence of 20 μM of MV with the addition of 0.2 mM of ADP and 2 mM of NaH_2_PO_4_; the uncoupled transfer rate was measured in the presence of 20 μM of MV with the addition of 1 μM of gramicidin D. Data are given as mean values ± SD (n = 6). Similar results were obtained with thylakoids isolated from plants of three independent plantings. Student’s t-test (with adjustment by Holm–Bonferroni method) is used for multiple comparisons among three groups for basal electron transfer type (no mafenide, 0.1 mM of mafenide, and 4 mM of HCO_3_^–^) and five groups for coupled/uncoupled electron transfer type (no mafenide, 0.1–0.5 mM of mafenide, and 4 mM of HCO_3_^–^). Values not connected by the same letter are significantly different (Student’s t-test, P < 0.001).*

*n.d., not determined.*

The lower electron transport rates in all cases (basal, coupled, and uncoupled) as compared with those of pea thylakoids (Fedorchuk et al., 2018) probably reflect the features of organization of thylakoid membranes of *Arabidopsis*. Previously, it was demonstrated that the thylakoid membranes from *Arabidopsis* were completely dissolved at significantly lower concentrations of detergents than the thylakoid membranes from pea plants (Ignatova et al., 2011), suggesting more loose structure of *Arabidopsis* thylakoid membranes. This difference was clearly illustrated by confocal images of isolated protoplasts from *Arabidopsis* and pea plants (Ignatova et al., 2011). Due to the difference, when working with *Arabidopsis*, much higher Mg^2+^ concentrations in isolation media were required for isolation of thylakoid membranes enriched with either PSI or PSII. It is noteworthy that the absolute rate of electron transfer in *Arabidopsis* thylakoids under coupled with ATP synthesis conditions (Table 1) was close to that shown in Casazza et al. (2001). An increase of the electron transfer rate under uncoupled conditions (Table 1) is also in line with the data of Casazza et al. (2001).

Table 1 shows that mafenide at the concentration of 0.1 mM did not affect the rate of electron transport under uncoupling conditions, i.e., when lumen pH has no effect on the electron transfer along PETC from water to MV, while the concentration of 0.25 mM and higher mafenide inhibited this rate. Since the absence of the effect of any substances on the uncoupled transport indicates the absence of the direct interaction with PETC components, the concentration of mafenide of 0.1 mM was used to study the effect of bicarbonate on PP rate in *Arabidopsis* thylakoids in further experiments. It may be noted that in pea thylakoids, mafenide affected the rate of electron transport only at concentrations higher than 2 mM (Fedorchuk et al., 2018). This difference between pea and *Arabidopsis* thylakoids could also be attributed to the different structures of thylakoid membranes in these plant species (Ignatova et al., 2011).

The addition of 4 mM of HCO_3_^–^ to the thylakoid suspension did not affect uncoupled and basal electron transport rates (Table 1). The absence of effect on the uncoupled electron transport rates suggested that HCO_3_^–^ had no direct influence on the electron transfer along PETC. In particular, it meant that HCO_3_^–^ addition at this concentration does not affect the electron transfer between Q_*A*_ and Q_*B*_ on the acceptor side of PSII. Thus, bicarbonate-dependent regulation of electron transfer between quinones on the acceptor side of PSII (“bicarbonate effect”) (Wydrzynski and Govindjee, 1975; Tikhonov et al., 2018) was not exhibited under used experimental conditions. The unaltered rate of basal transport indicates that under these experimental conditions, the addition of 4 mM of HCO_3_^–^ does not affect the already established connection of electron transport with proton gradient across the thylakoid membrane. At the same time, the addition of HCO_3_^–^ partly suppressed the coupled electron transfer rate (Table 1). The above results correspond well with our data obtained with pea thylakoids (Fedorchuk et al., 2018).

**FIGURE 1:**
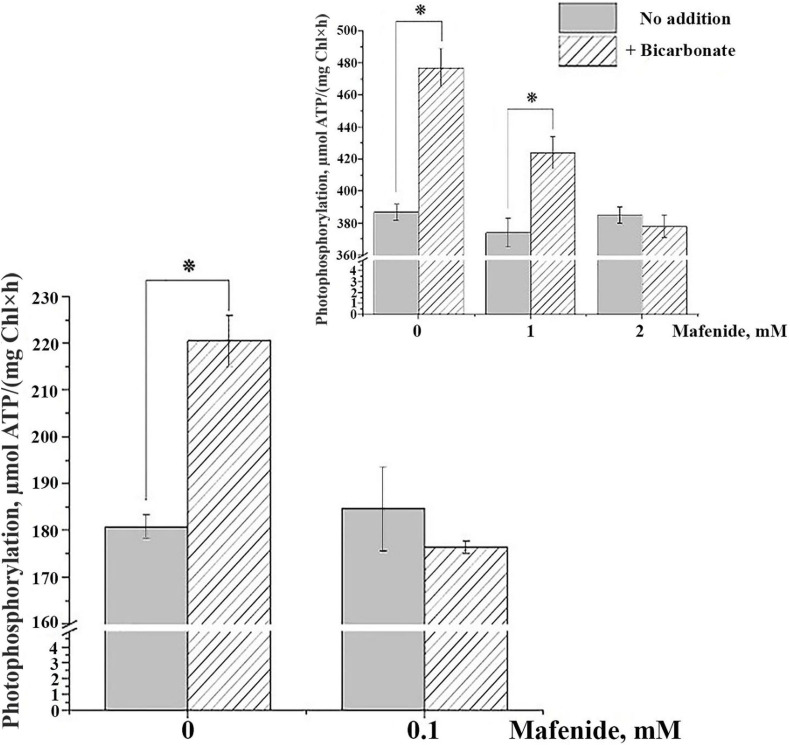
Effect of bicarbonate and mafenide on the rate of photophosphorylation (PP) in thylakoids from *Arabidopsis thaliana* plants (main panel) and from *Pisum sativum* (inset). The data for thylakoids from *P. sativum* are reproduced from Fedorchuk et al. (2018) with modifications. HCO_3_^–^ was present in the reaction mixture at concentration of 4 mM; mafenide was added at the concentrations indicated on the *Y*-axis. For detailed conditions of the experiments, see “Materials and Methods” section. Data are given as mean values ± SD (*n* = 6). Similar results were obtained with thylakoids isolated from plants of three independent plantings. * Statistically significant differences (*P* < 0.01).

In thylakoids isolated from *Arabidopsis* leaves, we observed an increase in PP rate in experiments with PMS as a cofactor when 4 mM of HCO_3_^–^ was added (Figure 1, main panel). This result is in total accordance with the data presented in studies with thylakoids from oat, spinach, and pea (Punnett and Iyer, 1964; Cohen and MacPeek, 1980; Onoiko et al., 2010; Fedorchuk et al., 2018). Mafenide at the concentration of 0.1 mM did not suppress the rate of PP in the absence of HCO_3_^–^; however, it completely revoked the stimulatory effect of HCO_3_^–^ on PP (Figure 1, main panel). The same effect was observed with pea thylakoids, although at higher concentrations of mafenide (Fedorchuk et al., 2018; Figure 1, inset). The increase in PP rate after addition of 4 mM of HCO_3_^–^ was observed also when only non-cyclic electron transport in the presence of MV occurs (not shown). Considering the decrease of coupled electron transport rate with MV in the presence of HCO_3_^–^ (Table 1), we can propose the emergence of additional process stimulating PP in the presence of HCO_3_^–^ that is non-coupled with electron transfer along PETC. Such stimulation of PP would be in good agreement with the higher P/e_2_ ratios that were obtained for non-cyclic phosphorylation in the pioneer study (Punnett and Iyer, 1964).

### Identification of Carbonic Anhydrase in Stromal Thylakoid Membranes

The results of our previous studies with pea thylakoids as well as the data of this study with *Arabidopsis* thylakoids indicated an involvement of CA in stimulation of PP by HCO_3_^–^. Taking this into account, and the fact that ATP-synthase complex performing PP is situated almost exclusively in STM, the identification of CA in these membranes was undertaken. The presence of a protein with CA activity has already been confirmed in such membranes isolated from both pea and *Arabidopsis* plants (Ignatova et al., 2006, 2011).

**FIGURE 2:**
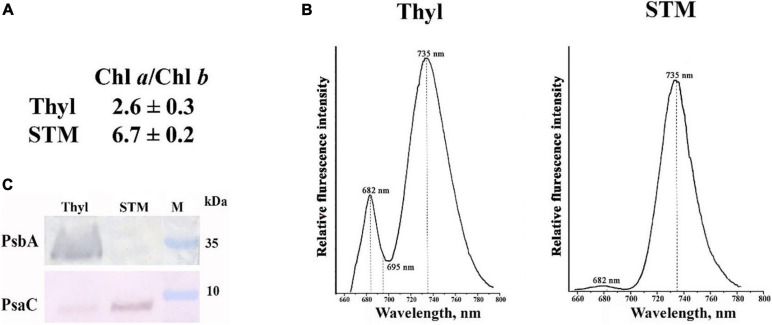
Characteristics of thylakoids (Thyl) and stromal thylakoid membranes (STM) isolated from *Arabidopsis thaliana* leaves. Chl *a*/Chl *b* ratio **(A)**; low-temperature chlorophyll fluorescence spectrum of Thyl and STM **(B)**; Western blot analysis of the proteins in Thyl and STM with antibodies against PsbA and PsaC, the major proteins of photosystem II (PSII) and photosystem I (PSI), respectively **(C)**. M, molecular mass markers (kDa).

STM preparations are where almost all PSI complexes and ATP-synthase complexes are situated. The isolation of STM is described in “Materials and Methods” section, and we characterized STM purity using three approaches. Firstly, the Chl *a*/Chl *b* ratio in STM was found to be close to 6.7 (Figure 2A), which is representative of the thylakoid membranes containing mainly PSI (Lam et al., 1984; Yamamoto et al., 2013), whereas in isolated whole thylakoids, it was about 2.6 (Figure 2A), which is typical for thylakoid membranes containing PSI, PSII, and light-harvesting complexes (Xu et al., 2001). Secondly, low-temperature Chl *a* fluorescence spectrum of these preparations had a pronounced peak at 735 nm (Figure 2B), which is the fluorescence maximum of Chl molecules bound to PSI (Cederstrand and Govindjee, 1966), while almost no fluorescence peaks were detected at 682/685 and 695 nm, i.e., of Chl molecules bound to PSII (Lam et al., 1984; Yamamoto et al., 2013). Thirdly, Western blot analysis has revealed a high content of PsbA, a major protein of PSII, in whole thylakoids and complete absence of PsbA in STM preparations (Figure 2C); at the same time, the band of PsaC, one of the major proteins of PSI, was well pronounced in STM preparations (Figure 2C). It indicates that STM preparations were vastly enriched with PSI and did not contain PSII complexes.

**FIGURE 3:**
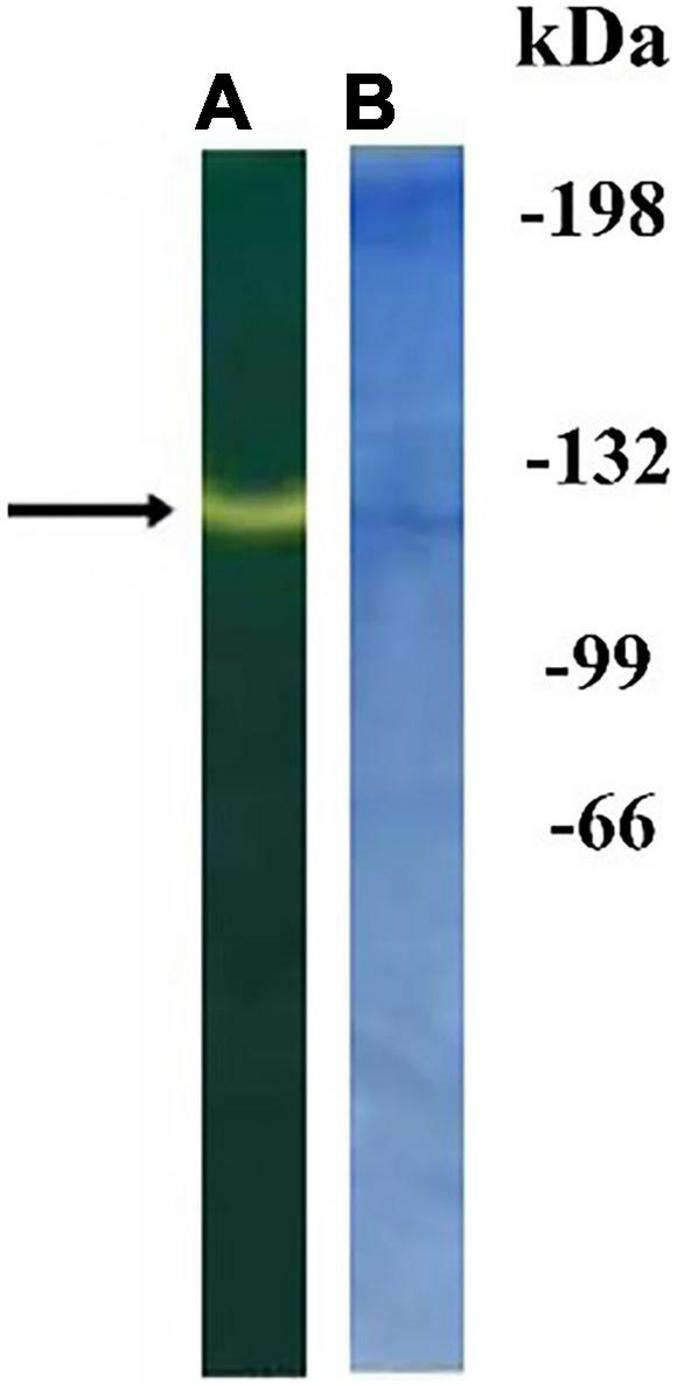
Non-denaturing electrophoresis of proteins from thylakoid membranes (STM) obtained after affinity chromatography on agarose with mafenide: gel stained with bromothymol blue **(A)**; the same gel stained with Coomassie G-250 **(B)**. The numbers on the right are the positions of the marker proteins with indicated molecular masses in kDa. The arrow shows the region of carbonic anhydrase (CA) activity.

STM preparations were broken down using high concentration of Triton X-100, and proteins were precipitated with acetone. After solubilization, they were purified by affinity chromatography followed by non-denaturing PAAG electrophoresis (see “Materials and Methods” section). The protein band showing CA activity was revealed on bromothymol blue stained gel as a change of color of bromothymol blue when fed with CO_2_-saturated water (Figure 3).

**FIGURE 4:**
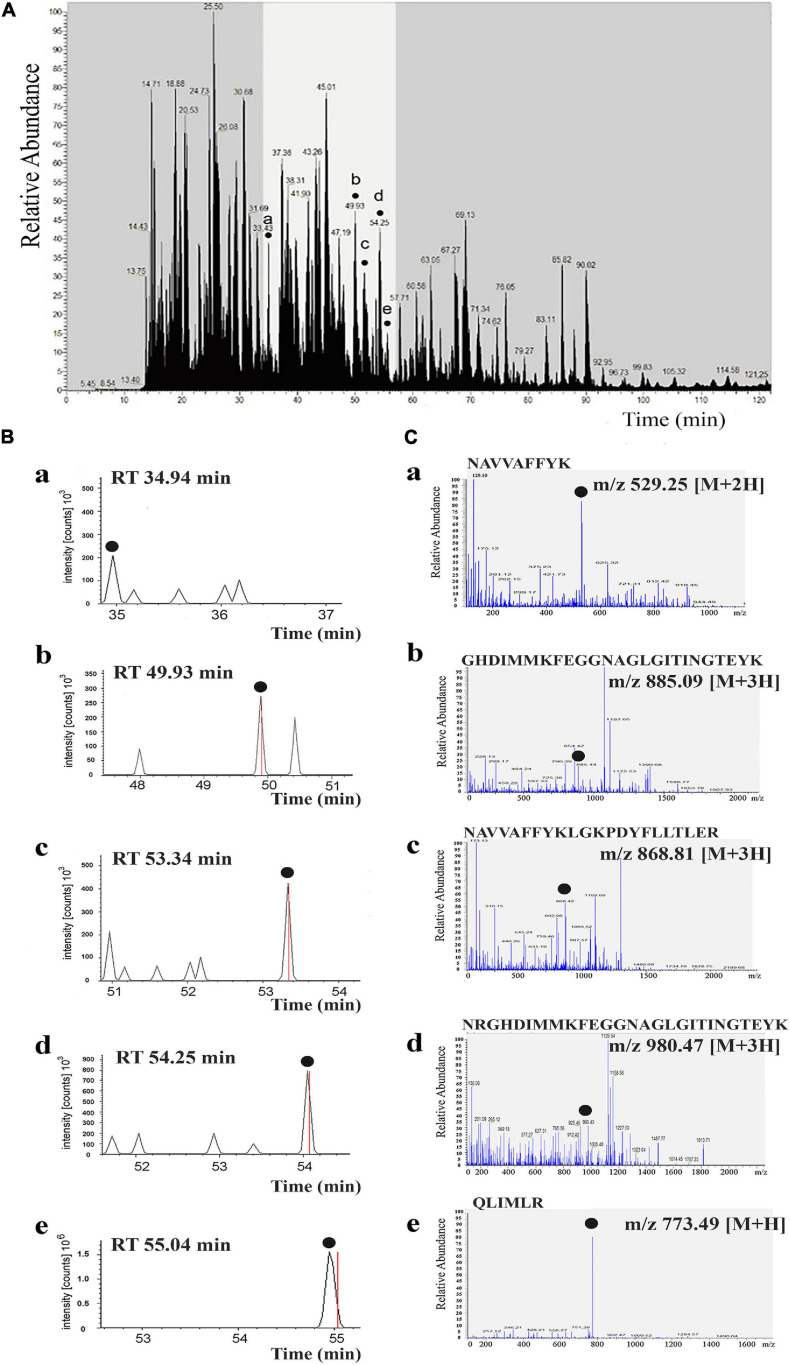
Tandem mass spectra of identified peptides of αCA5 from *Arabidopsis thaliana*. Liquid chromatography–tandem mass spectrometry (LC-MS/MS) chromatogram from trypsin digestion of the protein band with carbonic anhydrase (CA) activity after non-denaturing electrophoresis of thylakoid membranes (STM). Five peptides of αCA5 were identified, shown by dots a–e, denoting peptide peaks. These peaks correspond to amino acid sequences in the predicted primary structure of the product of the *At1g08065* gene, encoding αCA5 from *A. thaliana* **(A)**. Extracted ion chromatograms of five peptides (plots a–e) corresponding to the retention time (RT) of each peptide on the LC-MS/MS chromatogram **(B)**. MS/MS spectra obtained in data-dependent mode with an automatic switch between a full scan and up to seven data-dependent MS/MS scans. MS/MS spectra corresponding to each extracted ion chromatogram of five peptides are shown. The amino acid sequence and *m*/*z* ratio of each peptide is indicated at the top of each panel **(C)**.

The corresponding zone of the gel was cut out and used for preparation of trypsin-digested protein lysates. Analysis of obtained lysates was performed by LC-MS/MS (see “Materials and Methods” section) in triplicate (three samples of lysates). These protein samples containing 1 pmol of total protein were analyzed, but the target protein content in the band was only 110 fmol. Acquisition of tandem mass spectra during chromatographic analysis of the samples resulted in identifying an average of five peptides per target protein in the band, as shown in Figure 4A, peaks a–e. A series of representative MS/MS spectra are shown and provide evidence for the identification and localization of the measured peptide ions. The b- and y-ions are the main product ions formed when the original peptide is split at a peptide bond between two amino acids. For a given peptide sequence, the b-ions are the product when the charge is retained on the N-terminus (i.e., at the beginning of the sequence) and the y-ions the product when the charge is retained at the C-terminus (i.e., at the end of the sequence). For example, for the sequence NAVVAFFYK (Supplementary Figure 4a), the b3- and y3-ions correspond to splitting the peptide after the third amino acid: b3 = NAV and y3 = KYFFAV (y-ions are written in reverse order). All the b-ions shown in red and y-ions in blue (Supplementary Figures 1, 2, 4a) are the evidence of this identification. Ions that have a neutral loss are shown in green (Supplementary Figure 2, 4a), regardless of whether they are b- or y-ions. The more fragments found for a given identification, the more likely it is to be correct.

The full list of all ions that correspond to a given identification of all five peptides are shown in the fragment that matches tables and fragment that matches spectrums found in Supplementary Figures 4a–e. The spectra were produced by using SEQUEST (Thermo Scientific Proteome Discoverer software) (Supplementary Figures 4a–e).

In all three samples, the target protein was present, and it was identified consistently and reproducibly. The peptides were eluted over a period of 120 min (300 nl/min). Peptides of the target protein were released in time period from 30 to 60 min, as highlighted in Figure 4B (plots a–e). The scan range of the instrument was *m*/*z* 300–1,400, so considering singly, doubly, and triply charged peptides, the molecular mass sampling range is 300–2,000 Da. Data-dependent acquisition (DDA) mass spectrum was recorded on the (M + 2H)^2+^ ions at *m*/*z* 529.25 of a peptide NAVVAFFYK; the (M + 3H)^3+^ ions at *m*/*z* 885.09 of a peptide GHDIMMKFEGGNAGLGITINGTEYK; the (M + 3H)^3+^ ions at *m*/*z* 868.81 of a peptide NAVVAFFYKLGKPDYFLLTLER; the (M + 3H)^3+^ ions at *m*/*z* 980.47 of a peptide NRGHDIMMKFEGGNAGLGITINGTEYK; and the (M + H)^+^ ions at *m*/*z* 773.49 of a peptide QLIMLR from the αCA5 protein (Figure 4C, peaks **a–e**).

**FIGURE 5:**
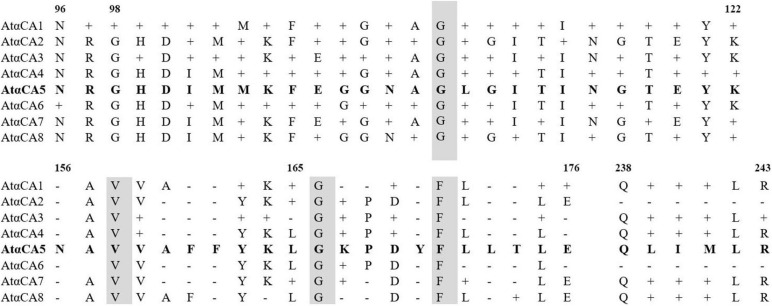
Comparison of the deduced amino acid sequences of αCA1, αCA2, αCA3, αCA4, αCA6, αCA7, and αCA8 with αCA5. Multiple alignment was performed with UniProt (https://www.uniprot.org/blast/). Gray–shaded boxes represent identical amino acids; dashes represent gaps in the alignment.

To confirm that the five unique peptides belong to αCA5, multiple sequence alignments of the amino acid sequences of the peptides detected by LC-MS/MS were compared with the sequences of other *Arabidopsis* αCAs, retrieved from The UniProt Consortium, 2021^1^. No significant alignments between the five unique peptides of αCA5 and the corresponding regions of amino acid sequences of other isoforms of αCAs were found (Figure 5).

Therefore, the sequences of the determined peptides were unique for the sequence of predicted αCA5 from the nucleotide sequence of the *At1g08065* gene. This result convincingly demonstrates that the protein with CA activity in STM of *A. thaliana* is indeed αCA5. No other CAs were detected in STM of *Arabidopsis* after LC-MS/MS of STM preparations.

### The Absence of Photophosphorylation Stimulation by Bicarbonate in Thylakoids From *Arabidopsis* Plants With Knocked-Out Gene Encoding αCA5

PP and its stimulation by bicarbonate were measured in thylakoids isolated from leaves of WT and αCA5-KO plants (see “Materials and Methods” section). Thylakoids of two lines of αCA5-KO plants were photochemically active since the rates of electron transfer under coupling and uncoupling conditions were significantly higher than the rate under basal conditions, as in thylakoids of WT plants; the addition of 0.1 mM of mafenide also did not affect these rates noticeably (Supplementary Table 1).

**FIGURE 6:**
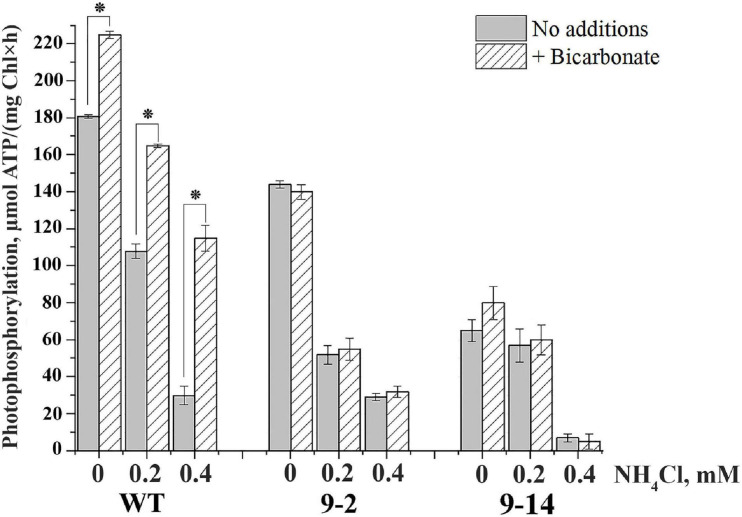
The effect of bicarbonate and NH_4_Cl on the rate of photophosphorylation in thylakoids isolated from leaves of wild-type (WT) *Arabidopsis thaliana* and two lines of αCA5-KO,“9–2” and “9–14” lines. HCO_3_^–^ was present in reaction mixture at concentration of 4 mM; NH_4_Cl was added at concentrations indicated on the *Y*-axis. For detailed conditions of the experiments, see “Materials and Methods” section. Data are given as mean values ± SD (*n* = 6). Similar results were obtained with thylakoids isolated from plants of three independent plantings. * Statistically significant differences (*P* < 0.01).

The rate of PP was lower in thylakoids of two lines of αCA5-KO plants in comparison with WT thylakoids (Figure 6). Unlike the stimulation of PP by addition of bicarbonate in thylakoids of WT plants, bicarbonate had no effect on the PP rate in thylakoids of αCA5-KO plants (Figure 6).

Cohen and MacPeek (1980) showed that bicarbonate alleviated the inhibitory effect of ammonium, which functions in thylakoids as an uncoupler, suppressing ATP synthesis. We observed a pronounced difference in PP stimulation by bicarbonate between thylakoids from WT and two lines of αCA5-KO plants in the presence of NH_4_Cl in the medium. Submillimolar concentrations of ammonium, 0.2 and 0.4 mM, substantially suppressed PP in the absence of HCO_3_^–^ in thylakoids of WT and two lines of αCA5-KO plants (Figure 6). These NH_4_Cl concentrations were intentionally lower than 10–30 mM, usually used to suppress PP completely. Addition of bicarbonate significantly alleviated the inhibitory effect of NH_4_Cl on ATP synthesis in thylakoids of WT plants, increasing PP rate. It was noted that the addition of bicarbonate more significantly stimulated PP in preparations of thylakoids of WT with initially lower PP rates (not shown). It can be seen that the stimulation of PP by bicarbonate was higher at higher concentrations of NH_4_Cl, i.e., under more significant PP inhibition by uncoupler (Figure 6). Similar data were previously obtained with thylakoids from pea plants (Fedorchuk et al., 2018). In thylakoids of two lines of αCA5-KO plants, PP stimulation by bicarbonate was detected neither in the absence nor in the presence of NH_4_Cl (Figure 6).

Thus, we can conclude that the stimulation of PP by bicarbonate depends on the presence of αCA5 in *Arabidopsis* thylakoids.

## Discussion

This study demonstrates that the stimulation of PP by adding HCO_3_^–^ to suspension of thylakoids isolated from WT *Arabidopsis* plants is absent in the presence of mafenide, a CA inhibitor, pointing out the involvement of CA in this stimulation. The specific CA responsible for this stimulation in *Arabidopsis* thylakoids was identified as αCA5, the protein encoded by *At1g08065* gene. Revealing the presence of this CA in STM contributes significantly to the understanding of the location of αCA5 in a photosynthesizing cell and its function. αCA5-KO plants had no phenotype differences from the WT plants (Supplementary Figure 1). This is a quite common observation for mutants with knocked-out synthesis of only one CA: the mutants with suppressed synthesis of the most abundant stromal βCA did not show any phenotypic differences from the WT tobacco plants (Price et al., 1994). The reasons for this phenomenon are still unclear, but it is possible that the absence of the enzymes can be compensated through some mechanisms at the whole-plant level. However, the effect of the absence of CA can be observed in studies with isolated structures.

In pea thylakoids, it was shown that the CA activity in STM was equally inhibited both by a membrane-permeable CA inhibitor, ethoxzolamide, and by a CA inhibitor, which is hardly able to pass through biological membranes, acetazolamide (Ignatova et al., 2006, 2011). A soluble inhibitor of CA, mafenide, also decreases CA activity of thylakoids (Fedorchuk et al., 2018). These results together with the results of the present study (Figure 1) allowed us to assume that αCA5 is located on the stromal surface of STM, where it is equally accessible to acetazolamide, ethoxzolamide, and water-soluble mafenide. The absence of PP stimulation by bicarbonate in two lines of αCA5-KO mutants (Figure 6) and the confirmation of the location of αCA5 in STM imply that the stimulatory effect of bicarbonate on PP is caused by dehydration of added HCO_3_^–^, catalyzed by αCA5 located in the stroma-exposed thylakoid regions.

We propose a tentative hypothesis of mechanism of αCA5 involvement in the stimulation of PP in thylakoids in the presence of bicarbonate excess. Since αCA5 and ATPase are both located in STM, we assume that bicarbonate dehydration by αCA5 accompanied by the consumption of protons leads to pH increase near the stromal surface of STM. This should result in ΔpH increase across the thylakoid membrane and therefore in the increased capability to perform ATP synthesis. The stimulation of ATP synthesis by an increase of ΔpH was described in detail in the classic studies (Schuldiner et al., 1972; Pick et al., 1974). However, an increase in pH outside thylakoids may not be the only reason to increment ΔpH across the membrane. Since the bicarbonate dehydration occurs at the surface of STM, the concentration of CO_2_ should increase at this location. It is known that the main barrier for CO_2_ molecules to cross lipid membranes is near-membrane unstirred layers, while membranes itself are easily permeable for these molecules (Missner et al., 2008). It is quite possible that some of the CO_2_ molecules that emerged enter these layers, from which they easily penetrate the lumen of the thylakoids. In the lumen, these molecules can be hydrated with proton release, resulting in the proton concentration increase in this compartment. The CO_2_ hydration can be accelerated by a soluble CA of β-family that was found to be located in the lumen of both pea and *Arabidopsis* thylakoids (Rudenko et al., 2007; Fedorchuk et al., 2014). New protons, which appear in the lumen, contribute in the increase of ΔpH and subsequently in the increase of PP rate. The process of CO_2_ hydration should proceed with higher probability under phosphorylating conditions, when proton concentration in the lumen decreased due to their outflow through ATP synthase. This leads to lumen pH increase almost by one unit as compared with basal conditions (Tikhonov, 2013), which formally corresponds to a 10-fold decrease in the proton concentration. Hereby, CO_2_ hydration process provides just under coupling conditions more perceptible increase in the proton concentration in the lumen. This explains the decrease in the linear electron transport rate just under coupling conditions in response to addition of HCO_3_^–^ to thylakoid suspension (Table 1), while under basal conditions, when the lumen pH is considerably lower than under coupling conditions, the flow of CO_2_ to the lumen is hardly possible, and the rate of electron transport does not change (Table 1). The stimulation of PP rate in the media with pH lower than 8.0 (Punnett and Iyer, 1964; Cohen and MacPeek, 1980; Onoiko et al., 2010) can also be related to the increase in ΔpH. Such increase helps to achieve a threshold for the conformational changes promoting the activity of coupling factors CF1 that leads to an increase of PP rate (Pick et al., 1974). The effect of HCO_3_^–^ addition on the components of *pmf* in isolated thylakoids in the absence and presence of mafenide, as well as the values of ΔΨ and ΔpH in leaves of WT plants and αCA5-KO mutant plants, will be evaluated in future studies, using the measurements of characteristics of electrochromic shift.

The proposed rise in ΔpH across thylakoid membrane with αCA5 involvement can also explain the greater stimulation of the PP by bicarbonate addition in the presence of ammonium (Figure 6). The uncoupling effect of ammonium results mainly from binding of protons, pumped by PETC into the lumen, by NH_3_ molecules, which, being neutral, are capable of easily passing through the thylakoid membrane to the lumen space, preventing the consumed protons from being used in PP. The transformation of NH_3_ to NH_4_^+^ promotes the flow of new NH_3_ molecules from the outer medium into the lumen along a concentration gradient. Therefore, outside the thylakoid membrane, NH_4_^+^ is deprotonated to compensate for the lack of NH_3_, and this additionally decreases ΔpH and consequently PP rate. Thus, the release of protons on the stromal side of the membrane due to both the deprotonation of NH_4_^+^ and the functioning of ATP synthase takes place. The activity of αCA5 reduces local acidification near CF1 owing to consumption of protons released as a result of both these processes and, therefore, enhances ΔpH across the thylakoid membrane and accordingly PP more efficiently than in the absence of ammonium.

What is the possible physiological significance of αCA5 at the stromal surface of thylakoid membranes for the processes occurring in chloroplasts *in vivo*? Illumination of plants increases pH of chloroplast stroma from 7.0 to 8.0 (Heldt et al., 1973; Wu and Berkowitz, 1992). It leads to an increase in HCO_3_^–^ concentration (at 400 ppm in air and t = 20°C) from 70 up to 700 μM and to possible stimulation of PP due to the activity of αCA5. In theory, the Calvin–Benson cycle requires an exact ATP/NADPH ratio of 1.5. However, this cycle can only operate sustainably at a higher ATP/NADPH ratio due to an inevitable dispersal of ATP, as it is required not only in the Calvin–Benson cycle reactions but also in many other chloroplast processes, such as metabolite transport and protein synthesis. Therefore, the additional ATP production is essential (Cruz et al., 2004). Stimulation of PP in the presence of bicarbonate, which in *Arabidopsis* chloroplasts is carried out with involvement of αCA5, can be a common feature of its homolog operation in all C3 higher plant species. Taking this into account, we can assume the role of CA in the regulation of ATP synthesis rate in bioenergetic membranes of other living organisms.

## Data Availability Statement

The datasets presented in this study can be found in online repositories. The names of the repository/repositories and accession number(s) can be found below: MassIVE repository (https://massive.ucsd.edu) using Massive ID: MSV000087020.

## Author Contributions

TF: substantial contributions to the conception and design of the work. IK: LS MS-MS spectra and analyses of obtained data. VO: ATP synthesis measurements. MB-M and VT: electron transport measurements. MB-M, NR, and BI: revising manuscript critically for important and unique content, final approval of the version. All authors have read, commented and corrected text and have expressed their approval with its contents.

## Conflict of Interest

The authors declare that the research was conducted in the absence of any commercial or financial relationships that could be construed as a potential conflict of interest.

## Publisher’s Note

All claims expressed in this article are solely those of the authors and do not necessarily represent those of their affiliated organizations, or those of the publisher, the editors and the reviewers. Any product that may be evaluated in this article, or claim that may be made by its manufacturer, is not guaranteed or endorsed by the publisher.

## Footnotes

1
https://www.uniprot.org/blast/

## Abbreviations

α CA5-KO: *Arabidopsis* plants with knocked-out *At1g08065* gene, encoding α-CA 5
CA: carbonic anhydrase
Chl: chlorophyll
DM: *N*-dodecyl - β -D-maltoside
LC-MS/MS: liquid chromatography–tandem mass spectrometry
MV: methyl viologen
PAAG: polyacrylamide gel
PETC: photosynthetic electron transfer chain
*pmf*: proton motive force
PP: photophosphorylation
PSI: photosystem I
PSII: photosystem II
qRT-PCR: quantitative reverse transcription PCR
STM: stromal thylakoid membranes
Thyl: thylakoid membranes
WT: wild-type.

## References

[B1] Avdeef A. Kearney D. L. Brown J. A. Chemotti A. R. (1982). Bjerrum plots for the determination of systematic concentration errors in titration data. *Anal. Chem.* 54 2322–2326. 10.1021/ac00250a041

[B2] Casazza A. P. Tarantino D. Soave C. (2001). Preparation and functional characterization of thylakoids from Arabidopsis thaliana. *Photosynth. Res.* 68 175–180. 10.1023/A:1011818021875 16228340

[B3] Cederstrand C. N. Govindjee (1966). Some properties of spinach chloroplast fractions obtained by digitonin solubilization. *Biochim. Biophys. Acta* 120 177–180. 10.1016/0926-6585(66)90294-9 5961103

[B4] Cohen W. S. Jagendorf A. T. (1972). Inhibition of energy-linked reactions in chloroplasts by polygalacturonate. *Arch. Biochem. Biophys.* 150 235–243. 10.1016/0003-9861(72)90031-8 4260397

[B5] Cohen W. S. MacPeek W. A. (1980). A proposed mechanism for the stimulatory effect of bicarbonate ions on ATP synthesis in isolated chloroplasts. *Plant Physiol.* 66 242–245. 10.1104/pp.66.2.242 16661413 PMC440574

[B6] Cruz J. A. Avenson T. J. Kanazawa A. Takizawa K. Edwards G. E. Kramer D. M. (2004). Plasticity in light reactions of photosynthesis for energy production and photoprotection. *J. Exp. Bot.* 56 395–406. 10.1093/jxb/eri022 15533877

[B7] Edwards L. J. Patton R. L. (1966). Visualization of carbonic anhydrase activity in polyacrylamide gels. *Stain Technol.* 41 333–334. 10.3109/10520296609116335

[B8] Fabre N. Reiter I. M. Becuwe-Linka N. Genty B. Rumeau D. (2007). Characterization and expression analysis of genes encoding alfa- and beta carbonic anhydrases in Arabidopsis. *Plant Cell Environ.* 30 617–629. 10.1111/j.1365-3040.2007.01651.x 17407539

[B9] Fedorchuk T. P. Opanasenko V. K. Rudenko N. N. Ivanov B. N. (2018). Bicarbonate-induced stimulation of photophosphorylation in isolated thylakoids: effects of carbonic anhydrase inhibitors. *Biol. Membr.* 35 34–41. 10.7868/S0233475518010048

[B10] Fedorchuk T. P. Rudenko N. N. Ignatova L. K. Ivanov B. N. (2014). The presence of soluble carbonic anhydrase in the thylakoid lumen of chloroplasts from Arabidopsis leaves. *J. Plant Physiol.* 171 903–906. 10.1016/j.jplph.2014.02.009 24913047

[B11] Graham D. Perry G. L. Atkins C. A. (1974). “In search of a role for carbonic anhydrase,” in *Photosynthesis Mechanisms of Regulation of Plant Growth*, eds Bieleski R. L. Ferguson A. R. Cresswell M. M. (Wellington: The Royal Society), 251–258.

[B12] Harris D. A. (1978). The interactions of coupling ATPases with nucleotides. *Biochim. Biophys. Acta* 463, 245–273. 10.1016/0304-4173(78)90002-2 147104

[B13] Heldt H. W. Werdan K. Milovancev M. Geller G. (1973). Alkalization of the chloroplast stroma caused by light-dependent proton flux into the thylakoid space. *Biochim. Biophys. Acta* 314 224–241. 10.1016/0005-2728(73)90137-0 4747067

[B14] Ignatova L. K. Rudenko N. N. Khristin M. S. Ivanov B. N. (2006). Heterogeneous origin of carbonic anhydrase activity of thylakoid membranes. *Biochem. (Mosc.)* 71 525–532. 10.1134/S0006297906050099 16732731

[B15] Ignatova L. K. Rudenko N. N. Mudrik V. A. Fedorchuk T. P. Ivanov B. N. (2011). Carbonic anhydrase activity in Arabidopsis thaliana thylakoid membrane and fragments enriched with PSI or PSII. *Photosynth. Res.* 110 89–98. 10.1007/s11120-011-9699-0 22006267

[B16] Lam E. Ortiz W. Malkin R. (1984). Chlorophyll a/b proteins of photosystem I. *FEBS Lett.* 168 10–14. 10.1016/0014-5793(84)80197-0

[B17] Lee A. H.-Y. Bastedo D. P. Youn J.-Y. Lo T. Middleton M. A. Kireeva I. (2018). Identifying pseudomonas syringae type III secreted effector function via a yeast genomic screen. *G3 (Bethesda)* 9 535–547. 10.1534/g3.118.200877 30573466 PMC6385969

[B18] Lichtenthaler H. K. (1987). “Chlorophylls and carotenoids: pigments of photosynthetic biomembranes,” in *Methods in Enzymology*, eds Colowick N. Kaplan N. (Amsterdam: Elsevier), 350–382. 10.1016/0076-6879(87)48036-1

[B19] Lu Y.-K. Stemler A. J. (2002). Extrinsic photosystem II carbonic anhydrase in maize mesophyll chloroplasts. *Plant Physiol.* 128 643–649. 10.1104/pp.010643 11842167 PMC148926

[B20] Malyan A. N. (2003). Interaction of oxyanions with thioredoxin-activated chloroplast coupling factor 1. *Biochim. Biophys. Acta* 1607 161–166. 10.1016/j.bbabio.2003.09.009 14670606

[B21] Missner A. Kügler P. Saparov S. M. Sommer K. Mathai J. C. Zeidel M. L. (2008). Carbon dioxide transport through membranes. *J. Biol. Chem.* 283 25340–25347. 10.1074/jbc.M800096200 18617525 PMC2533081

[B22] Nelson N. Nelson H. Racker E. (1972). Partial resolution of the enzymes catalyzing photophosphorylation. XI. Magnesium-adenosine triphosphatase properties of heat-activated coupling factor I from chloroplasts. *J. Biol. Chem.* 247 6506–6510. 4263197

[B23] Nishimura M. Ito T. Chance B. (1962). Studies on bacterial photophosphorylation. III. A sensitive and rapid method of determination of photophosphorylation. *Biochim. Biophys. Acta* 59 177–182. 14479975

[B24] Onda Y. Matsumura T. Kimata-Ariga Y. Sakakibara H. Sugiyama T. Hase T. (2000). Differential interaction of maize root ferredoxin:NADP + oxidoreductase with photosynthetic and non-photosynthetic ferredoxin isoproteins. *Plant Physiol.* 123 1037–1046. 10.1104/pp.123.3.1037 10889253 PMC59067

[B25] Onoiko E. B. Polishchuck A. V. Zolotareva E. K. (2010). The stimulation of photophosphorylation in isolated spinach chloroplasts by exogenous bicarbonate: the role of carbonic anhydrase. *Dopov. Nac. Akad. Nauk. Ukr.* 10 160–165.

[B26] Peter G. F. Thornber J. P. (1991). Biochemical composition and organization of higher plant photosystem II light-harvesting pigment-proteins. *J. Biol. Chem.* 266 16745–16754. 1885603

[B27] Pick U. Rottenberg H. Avron M. (1974). The dependence of photophosphorylation in chloroplasts on ΔpH and external pH. *FEBS Lett.* 48 32–36. 10.1016/0014-5793(74)81055-0 4430371

[B28] Podorvanov V. V. Zolotareva E. K. Chernoshtan A. A. (2005). Role of bicarbonate in light-dependent proton uptake in isolated chloroplasts. *Fiziol. Biokhim. Kul’t. Rast.* 37 326–331.

[B29] Price G. D. von Caemmerer S. Evans J. R. Yu J.-W. Lloid J. Oja V. (1994). Specific reduction of chloroplast carbonic anhydrase activity by antisense RNA in transgenic tobacco plants has a minor effect on photosynthetic CO2 assimilation. *Planta* 193 331–340. 10.1007/BF00201810

[B30] Pronina N. A. Allakhverdiev S. I. Kupriyanova E. V. Klyachko-Gurvich G. L. Klimov V. V. (2002). Carbonic anhydrase in subchloroplast particles of pea plants. *Russ. J. Plant Physiol.* 49 303–310. 10.1023/A:1015589215862

[B31] Punnett T. Iyer R. V. (1964). The enhancement of photophosphorylation and the hill reaction by carbon dioxide. *J. Biol. Chem.* 239 2335–2339. 14209965

[B32] Rudenko N. N. Ignatova L. K. Ivanov B. N. (2007). Multiple sources of carbonic anhydrase activity in pea thylakoids: soluble and membrane-bound forms. *Photosynth. Res.* 91 81–89. 10.1007/s11120-007-9148-2 17347907

[B33] Rudenko N. N. Ignatova L. K. Kamornitskaya V. B. Ivanov B. N. (2006). Pea leaf thylakoids contain several carbonic anhydrases. *Dokl. Biochem. Biophys.* 408 155–157. 10.1134/S1607672906030136 16913418

[B34] Schägger H. von Jagow G. (1987). Tricine-sodium dodecyl sulfate-polyacrylamide gel electrophoresis for the separation of proteins in the range from 1 to 100 kDa. *Anal. Biochem.* 166 368–379. 10.1016/0003-2697(87)90587-2 2449095

[B35] Schuldiner S. Rottenberg H. Avron M. (1972). Membrane potential as a driving force for ATP synthesis in chloroplasts. *FEBS Lett.* 28 173–176. 10.1016/0014-5793(72)80704-X 11946850

[B36] Scopes R. K. (1987). *Protein Purification: Principles and Practice.* New York, NY: Springer.

[B37] Swader J. A. Jacobson B. S. (1972). Acetazolamide inhibition of photosystem II in isolated spinach chloroplasts. *Phytochemistry* 11 65–70. 10.1016/S0031-9422(00)89968-9

[B38] Tikhonov A. N. (2013). pH-Dependent regulation of electron transport and ATP synthesis in chloroplasts. *Photosynth. Res.* 116 511–534. 10.1007/s11120-013-9845-y 23695653

[B39] Tikhonov K. Shevela D. Klimov V. Messinger J. (2018). Quantification of bound bicarbonate in photosystem II. *Photosynthetica* 56 210–216. 10.1007/s11099-017-0758-4

[B40] The UniProt Consortium (2021). UniProt: the universal protein knowledgebase in 2021. *Nucleic Acids Res.* 49, D480–D489. 10.1093/nar/gkaa1100 33237286 PMC7778908

[B41] Wu W. Berkowitz G. A. (1992). Stromal pH and photosynthesis are affected by electroneutral K + and H + exchange through chloroplast envelope ion channels. *Plant Physiol.* 98 666–672. 10.1104/pp.98.2.666 16668693 PMC1080242

[B42] Wydrzynski T. Govindjee (1975). A new site of bicarbonate effect in photosystem II of photosynthesis: evidence from chlorophyll fluorescence transients in spinach chloroplasts. *Biochim. Biophys. Acta* 387 403–408. 1125295 10.1016/0005-2728(75)90121-8

[B43] Xu H. Vavilin D. Vermaas W. (2001). Chlorophyll b can serve as the major pigment in functional photosystem II complexes of cyanobacteria. *Proc. Natl. Acad. Sci. U. S. A.* 98 14168–14173. 10.1073/pnas.251530298 11717469 PMC61186

[B44] Yamamoto Y. Hori H. Kai S. Ishikawa T. Ohnishi A. Tsumura N. (2013). Quality control of photosystem II: reversible and irreversible protein aggregation decides the fate of Photosystem II under excessive illumination. *Front. Plant Sci.* 4:433. 10.3389/fpls.2013.00433 24194743 PMC3810940

