# Supplementary material for: Alpha Carbonic Anhydrase 5 Mediates Stimulation of ATP Synthesis by Bicarbonate in Isolated Arabidopsis Thylakoids
Source: Front Plant Sci. 2021 Aug 26;12:662082. doi: 10.3389/fpls.2021.662082 (PMC8427869; doi:10.3389/fpls.2021.662082)
Supplement: Supplementary file 1 [file Data_Sheet_1.PDF]

Supplementary Material

1 Supplementary Data

2 Supplementary Figures and Tables

2.1 Supplementary Figures

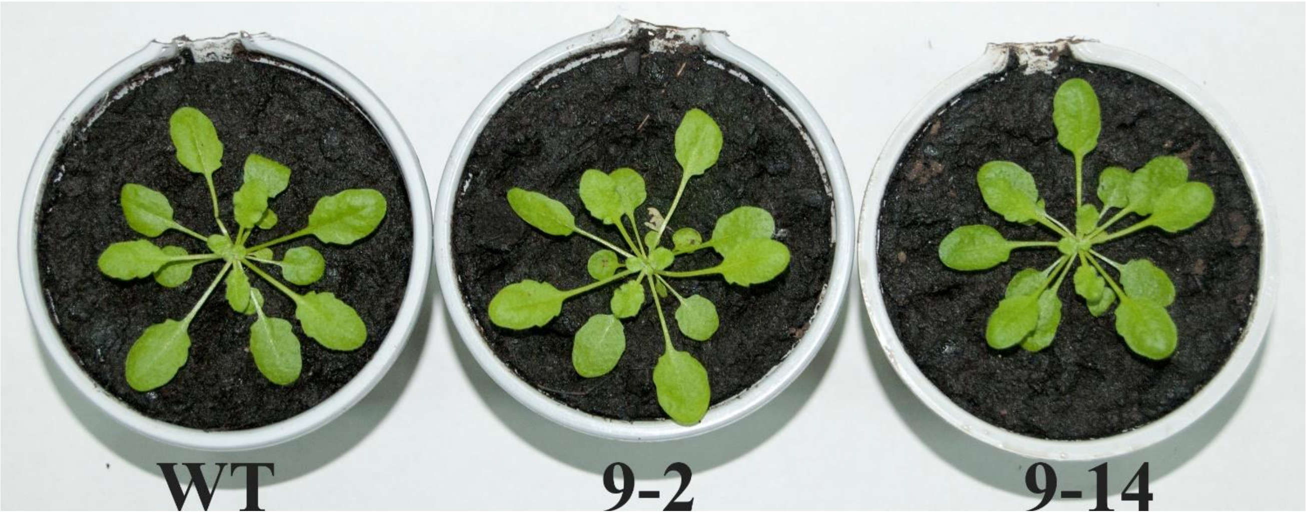

Supplementary Figure 1. *Arabidopsis thaliana* plant phenotypes of WT and of two lines with knocked out *At1g08065* gene, encoding αCA5 (“9-2” and “9-14”, respectively) after 40 days post germination growth (see Materials and Methods).

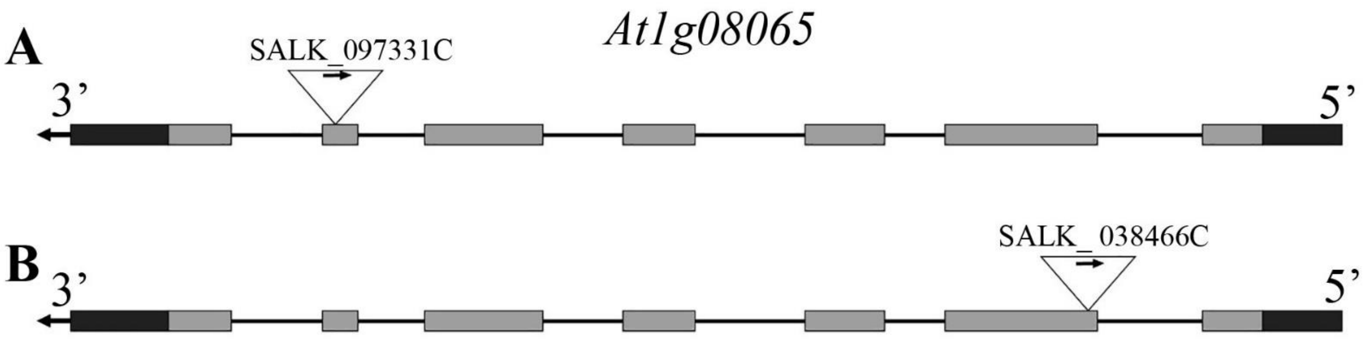

Supplementary Figure 2. Schematic representation of two T-DNA insertions in the *At1g08065* gene encoding αCA5. SALK_097331C is the position of the insertion in homozygous mutant plants "9-2" line (**A**) and SALK_038466C is the position of the insertion in homozygous mutant plants; "9-14" line **(B**). Gray boxes represent exons (black – *untranslated region*s, gray – *coding sequences*) and lines indicate introns. The triangles indicate T-DNA insertions; the arrows within the triangles indicate the orientation of the insertion.

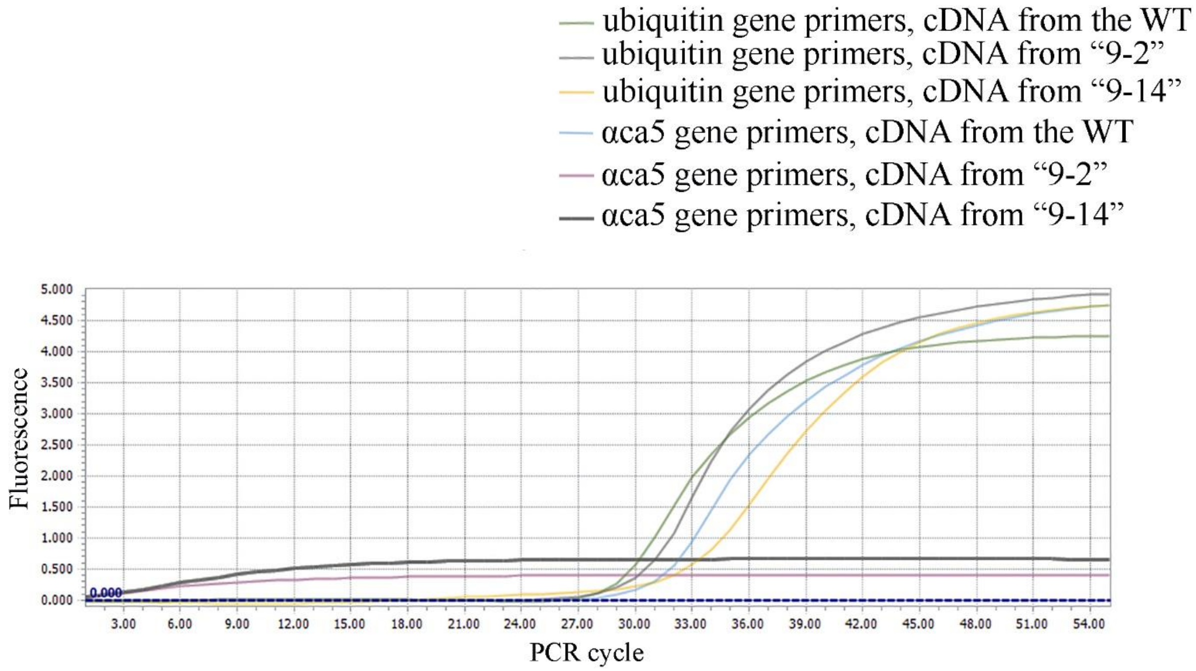

Supplementary Figure 3A. Amplification curves after qRT-PCR performed as described in Materials and Methods with cDNA from leaves of WT plants and αCA5-KO, “9-2” and “9-14” lines, *αca5* (*At1g08065*) gene primers and ubiquitin-encoding gene primers as a control.

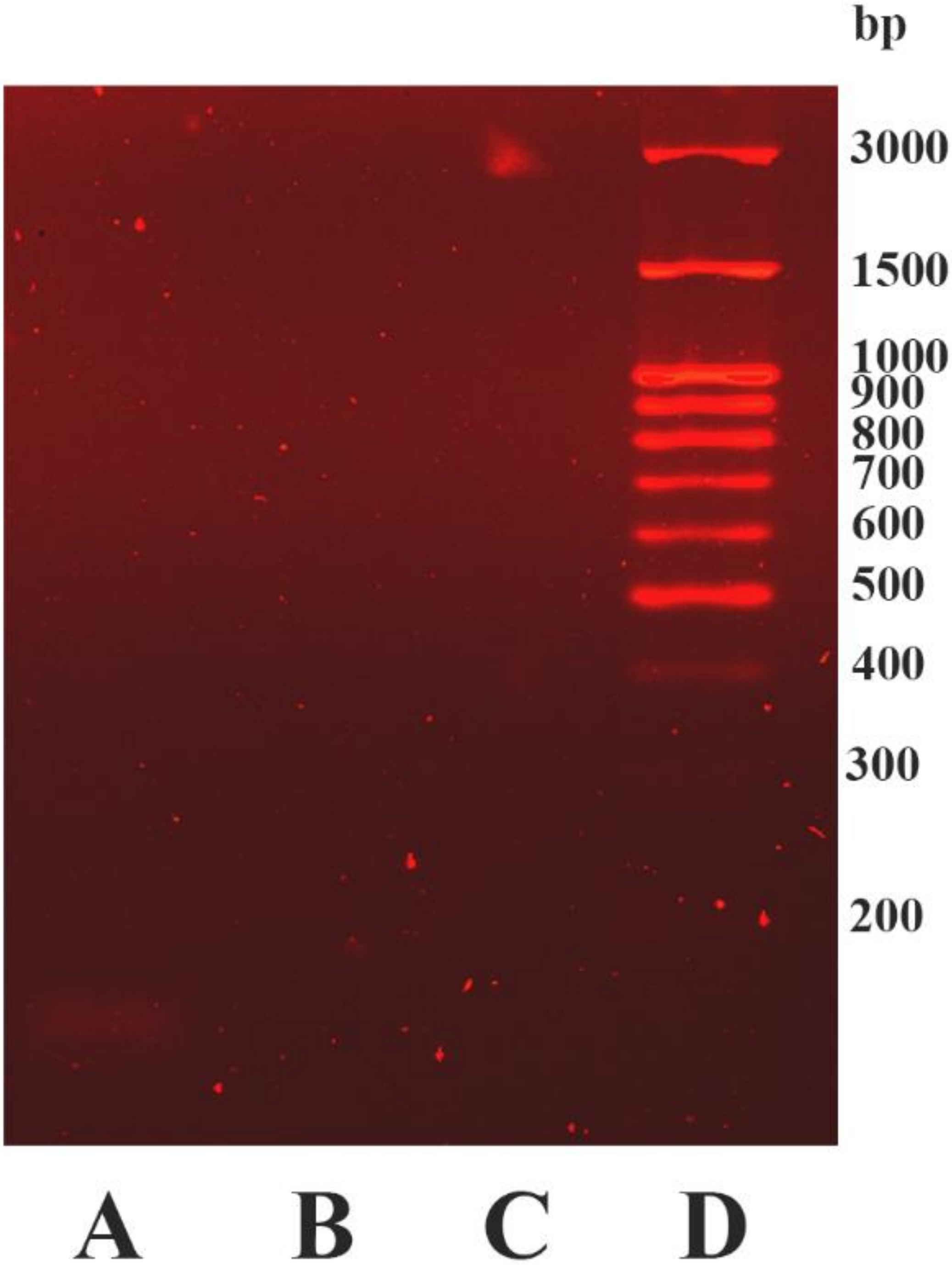

Supplementary Figure 3B. Electrophoresis of PCR products obtained with specific primer pair of the *At1g08065* gene. cDNA synthesis was performed with RNA from WT plants **(A)**, mutant line “9-2” **(B),** mutant line “9-14” **(C)**. DNA ladder with DNA fragments ranging from 200 bp to 3,000 bp (SibEnzyme, Russia) was used as a marker for electrophoresis **(D)**.

Supplementary Figure 4a. Additional evidence for the identification and localization of the NAVVAFFYK peptide determined. Ion fragment matches **(1)**, fragment matches spectrum **(2)**, monoisotopic ions **(3)**, and the data from FASTA file of αCA5 with sequence of the measured peptide colored in red **(4)** are shown.

1.

Fragment Matches

Value Type: Theo. Mass [Da]

Ion Series | Neutral Losses | Precursor Ions

#1	b^+	b^{2+}	Seq.	y^+	y^{2+}	#2
1	115.05020	58.02874	N			9
2	186.08732	93.54730	A	944.52402	472.76565	8
3	285.15573	143.08150	V	873.48690	437.24709	7
4	384.22415	192.61571	V	774.41849	387.71288	6
5	455.26126	228.13427	A	675.35007	338.17868	5
6	602.32967	301.66847	F	604.31296	302.66012	4
7	749.39809	375.20268	F	457.24455	229.12591	3
8	912.46142	456.73435	Y	310.17613	155.59170	2
9			K	147.11280	74.06004	1

2.

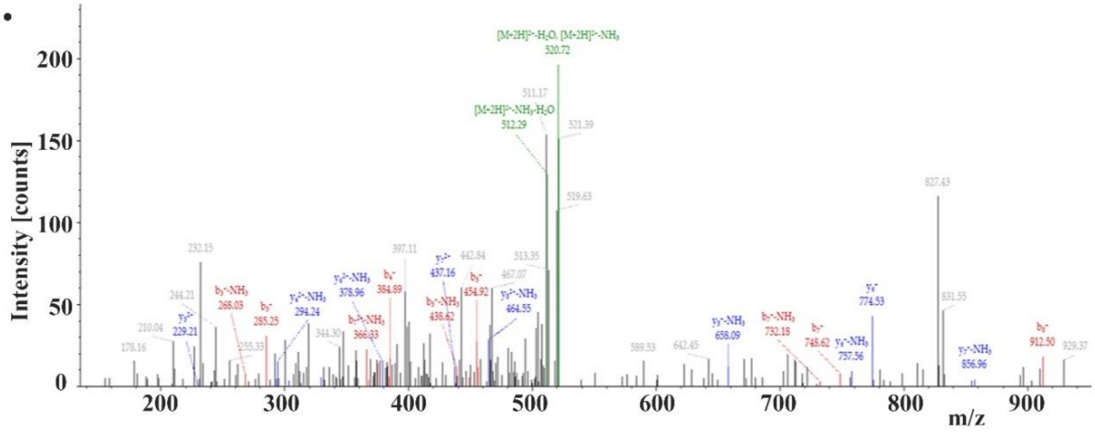

3.

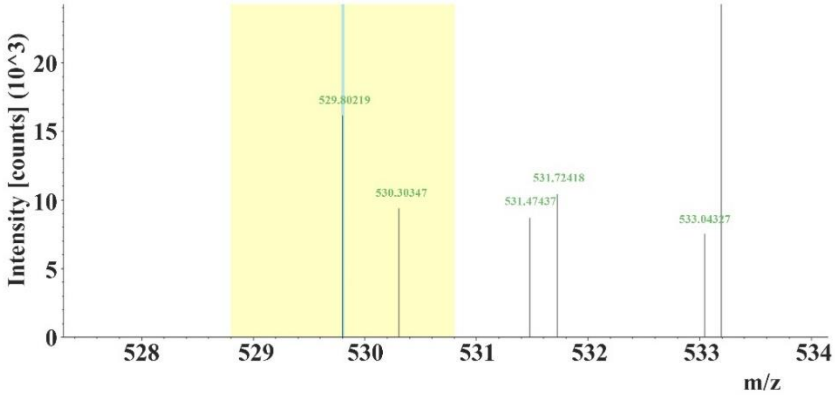

4.

>sp|F4HUC4|ATCA5_ARATH Alpha carbonic anhydrase 5 OS=Arabidopsis thaliana OX=3702 GN=ACA5 PE=3 SV=1

MKIPSIGYVFFLIFISITIVSSSPDHGEVEDETQFNYEKKGEKGPENWGRLKPEWAMCGK
GNMQSPIDLTDKRVLIDHNLGYLRSQYLPSNATIKNRGHDIMMKFEGGNAGLGITINGTE
YKLQQIHWHSPSEHTLNGKRFVLEEHMVHQSKDGRNAVVAFFYKLGKPDYFLLTLERYLK
RITDTHESQEFVEMVHPRTFGFESKHYYRFIGSLTTPPCSENVIWTISKEMRTVTLKQLI
MLRVTVHDQSNSNARPLQRKNERPVALYIPTWHSKLY

Supplementary Figure 4b. Additional evidence for the identification and localization of the GHDIMMKFEGGNAGLGITINGTE peptide determined. Ion fragment matches **(1)**, fragment matches spectrum **(2)**, monoisotopic ions **(3)**, and the data from FASTA file of αCA5 with sequence of the measured peptide colored in red **(4)** are shown.

1.

Fragment Matches

Value Type: Theo. Mass [Da]

Ion Series | Neutral Losses | Precursor Ions

#1	b^+	b^{2+}	b^{3+}	Seq.	y^+	y^{2+}	y^{3+}	#2
1	58.02874	29.51801	20.01443	G				25
2	195.08765	98.04746	65.70074	H	2596.25342	1298.63035	866.08932	24
3	310.11460	155.56094	104.04305	D	2459.19451	1230.10089	820.40302	23
4	423.19866	212.10297	141.73774	I	2344.16756	1172.58742	782.06071	22
5	554.23914	277.62321	185.41790	M	2231.08350	1116.04539	744.36602	21
6	685.27963	343.14345	229.09806	M	2100.04302	1050.52515	700.68586	20
7	813.37459	407.19093	271.79638	K	1969.00253	985.00490	657.00569	19
8	960.44300	480.72514	320.81919	F	1840.90757	920.95742	614.30737	18
9	1089.48560	545.24644	363.83338	E	1693.83915	847.42322	565.28457	17
10	1146.50706	573.75717	382.84054	G	1564.79656	782.90192	522.27037	16
11	1203.52853	602.26790	401.84769	G	1507.77510	754.39119	503.26322	15
12	1317.57145	659.28936	439.86200	N	1450.75363	725.88046	484.25606	14
13	1388.60857	694.80792	463.54104	A	1336.71071	668.85899	446.24175	13
14	1445.63003	723.31865	482.54819	G	1265.67359	633.34043	422.56272	12
15	1558.71409	779.86069	520.24288	L	1208.65213	604.82970	403.55556	11
16	1615.73556	808.37142	539.25004	G	1095.56807	548.28767	365.86087	10
17	1728.81962	864.91345	576.94473	I	1038.54660	519.77694	346.85372	9
18	1829.86730	915.43729	610.62728	T	925.46254	463.23491	309.15903	8
19	1942.95136	971.97932	648.32197	I	824.41486	412.71107	275.47647	7
20	2056.99429	1029.00078	686.33628	N	711.33080	356.16904	237.78178	6
21	2114.01576	1057.51152	705.34344	G	597.28787	299.14757	199.76747	5
22	2215.06343	1108.03536	739.02600	T	540.26640	270.63684	180.76032	4
23	2344.10603	1172.55665	782.04019	E	439.21873	220.11300	147.07776	3
24	2507.16936	1254.08832	836.39464	Y	310.17613	155.59170	104.06356	2
25				K	147.11280	74.06004	49.70912	1

2.

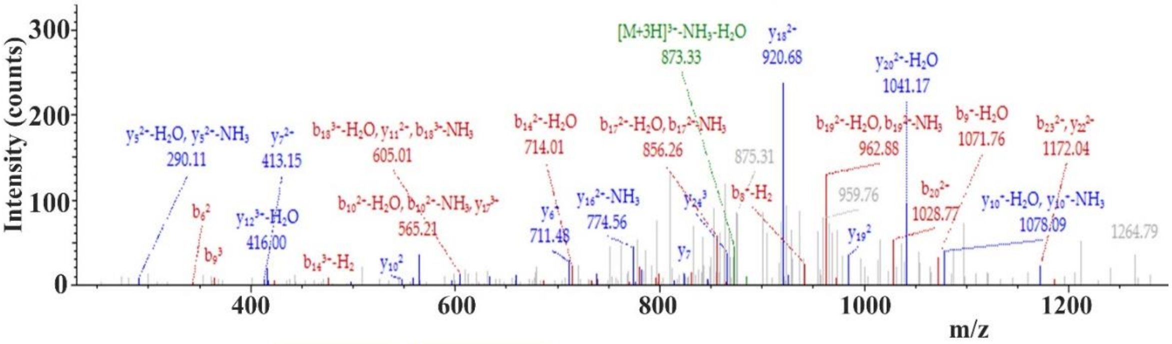

3.

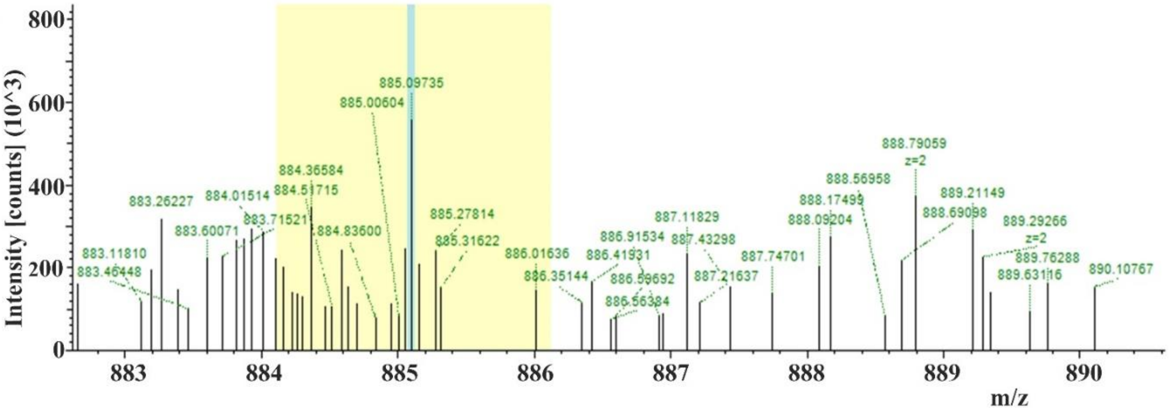

4.

>sp|F4IUC4|ATCA5_ARATH Alpha carbonic anhydrase 5 OS=Arabidopsis thaliana OX=3702 GN=ACA5 PE=3 SV=1

MKIPSIGYVFFLIFISITIVSSSPDHGEVEDETQFNYEKKGEKGPENWGRLKPEWAMCGK
GNMQSPIDLTDKRVLIDHNLGYLRSQYLPSNATIKNRGHDIMMKFEGGNAGLGITINGTE
YKLQQIHWHSPSEHTLNGKRFVLEEHMVHQSKDGRNAVVAFFYKLGKPDYFLLTLERYLK
RITDTHESQEFVEMVHPRTFGFESKHYYRFIGSLTTPPCSENVIWTISKEMRTVTLKQLI
MLRVTVHDQSNSNARPLQRKNERPVALYIPTWHSKLY

Supplementary Figure 4c. Additional evidence for the identification and localization of the NAVVAFFYKLGKPDYFLLTLER peptide determined. Ion fragment matches **(1)**, fragment matches spectrum **(2)**, monoisotopic ions **(3)**, and the data from FASTA file of αCA5 with sequence of the measured peptide colored in red **(4)** are shown.

1.

Fragment Matches

Value Type: Theo. Mass [Da]

Ion Series | Neutral Losses | Precursor Ions

#1	b+	b2+	b3+	Seq.	y+	y2+	y3+	#2
1	115.05020	58.02874	39.02159	N				22
2	186.08732	93.54730	62.70062	A	2490.37953	1245.69340	830.79803	21
3	285.15573	143.08150	95.72343	V	2419.34242	1210.17485	807.11899	20
4	384.22415	192.61571	128.74623	V	2320.27400	1160.64064	774.09619	19
5	455.26126	228.13427	152.42527	A	2221.20559	1111.10643	741.07338	18
6	602.32967	301.66847	201.44808	F	2150.16847	1075.58788	717.39434	17
7	749.39809	375.20268	250.47088	F	2003.10006	1002.05367	668.37154	16
8	912.46142	456.73435	304.82532	Y	1856.03165	928.51946	619.34873	15
9	1040.55638	520.78183	347.52364	K	1692.96832	846.98780	564.99429	14
10	1153.64044	577.32386	385.21833	L	1564.87336	782.94032	522.29597	13
11	1210.66191	605.83459	404.22549	G	1451.78929	726.39828	484.60128	12
12	1338.75687	669.88207	446.92381	K	1394.76783	697.88755	465.59413	11
13	1435.80963	718.40845	479.27473	P	1266.67286	633.84007	422.89581	10
14	1550.83658	775.92193	517.61704	D	1169.62010	585.31369	390.54488	9
15	1713.89990	857.45359	571.97149	Y	1054.59316	527.80022	352.20257	8
16	1860.96832	930.98780	620.99429	F	891.52983	446.26855	297.84813	7
17	1974.05238	987.52983	658.68898	L	744.46142	372.73435	248.82532	6
18	2087.13645	1044.07186	696.38367	L	631.37735	316.19231	211.13063	5
19	2188.18412	1094.59570	730.06623	T	518.29329	259.65028	173.43595	4
20	2301.26819	1151.13773	767.76091	L	417.24561	209.12644	139.75339	3
21	2430.31078	1215.65903	810.77511	E	304.16155	152.58441	102.05870	2
22				R	175.11895	88.06311	59.04450	1

2.

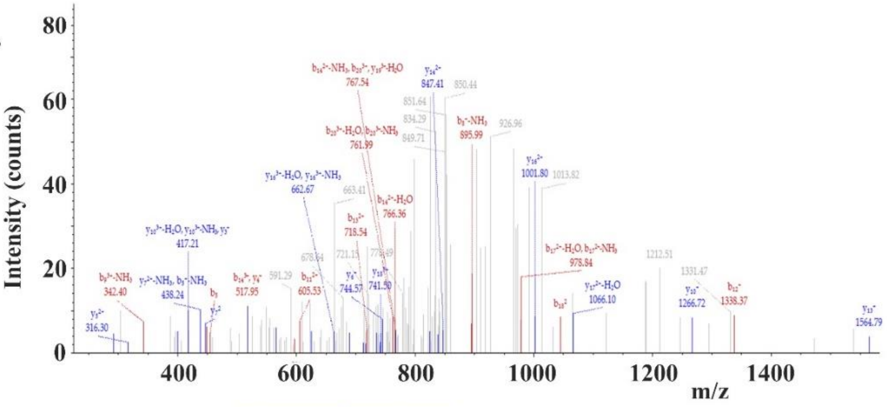

3.

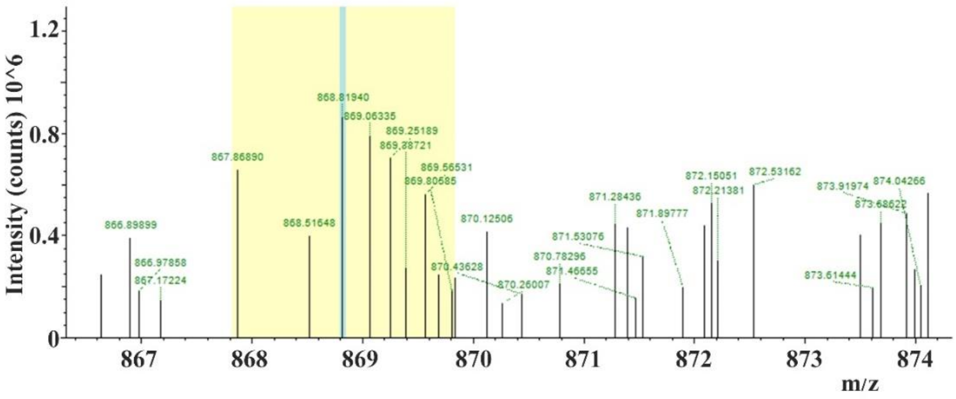

4.

>sp|F4HUC4|ATCA5_ARATH Alpha carbonic anhydrase 5 OS=Arabidopsis thaliana OX=3702 GN=ACA5 PE=3 SV=1
MKIPSIGYVFFLIFISITIVSSSPDHGEVEDETQFNYEKKGEKGPENWGRLKPEWAMCGK
GNMQSPIDLTDKRVLIDHNLGYLRSQYLPSNATIKNRGHDIMMKFEGGNAGLGITINGTE
YKLQQIHWHSPSEHTLNGKRFVLEEHMVHQSKDGRNAVVAFFYKLGKPDYFLLTLERYLK
RITDTHESQEFVEMVHPRTFGFESKHYYRFIGSLTTPPCSENVIWTISKEMRTVTLKQLI
MLRVTVHDQSNSNARPLQRKNERPVALYIPTWHSKLY

Supplementary Figure 4d. Additional evidence for the identification and localization of the NRGHDIMMKFEGGNAGLGITINGTEYK peptide determined. Ion fragment matches **(1)**, fragment matches spectrum **(2)**, monoisotopic ions **(3)**, and the data from FASTA file of αCA5 with sequence of the measured peptide colored in red **(4)** are shown.

1.

Fragment Matches

Value Type: Theo. Mass [Da]

Ion Series | Neutral Losses | Precursor Ions

#1	b^{+}	b^{2+}	b^{3+}	Seq.	y^{+}	y^{2+}	y^{3+}	#2
1	115.05020	58.02874	39.02159	N				27
2	271.15131	136.07930	91.05529	R	2825.37091	1413.18909	942.46182	26
3	328.17278	164.59003	110.06244	G	2669.26980	1335.13854	890.42812	25
4	465.23169	233.11948	155.74875	H	2612.24833	1306.62781	871.42096	24
5	580.25863	290.63296	194.09106	D	2475.18942	1238.09835	825.73466	23
6	693.34270	347.17499	231.78575	I	2360.16248	1180.58488	787.39234	22
7	824.38318	412.69523	275.46591	M	2247.07842	1124.04285	749.69766	21
8	971.41858	486.21293	324.47771	M-Oxidation	2116.03793	1058.52260	706.01749	20
9	1099.51354	550.26041	367.17603	K	1969.00253	985.00490	657.00569	19
10	1246.58196	623.79462	416.19884	F	1840.90757	920.95742	614.30737	18
11	1375.62455	688.31591	459.21303	E	1693.83915	847.42322	565.28457	17
12	1432.64602	716.82665	478.22019	G	1564.79656	782.90192	522.27037	16
13	1489.66748	745.33738	497.22734	G	1507.77510	754.39119	503.26322	15
14	1603.71041	802.35884	535.24165	N	1450.75363	725.88046	484.25606	14
15	1674.74752	837.87740	558.92069	A	1336.71071	668.85899	446.24175	13
16	1731.76898	866.38813	577.92785	G	1265.67359	633.34043	422.56272	12
17	1844.85305	922.93016	615.62253	L	1208.65213	604.82970	403.55556	11
18	1901.87451	951.44089	634.62969	G	1095.56807	548.28767	365.86087	10
19	2014.95858	1007.98293	672.32438	I	1038.54660	519.77694	346.85372	9
20	2116.00625	1058.50677	706.00694	T	925.46254	463.23491	309.15903	8
21	2229.09032	1115.04880	743.70162	I	824.41486	412.71107	275.47647	7
22	2343.13324	1172.07026	781.71593	N	711.33080	356.16904	237.78178	6
23	2400.15471	1200.58099	800.72309	G	597.28787	299.14757	199.76747	5
24	2501.20239	1251.10483	834.40565	T	540.26640	270.63684	180.76032	4

2.

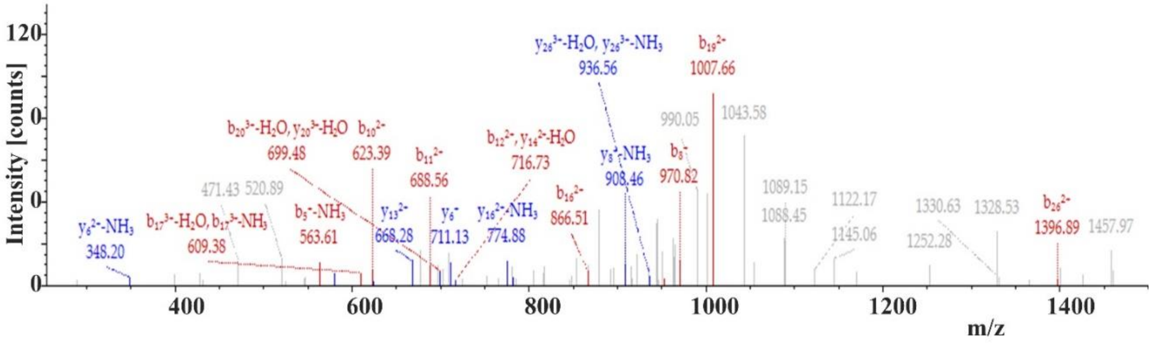

3.

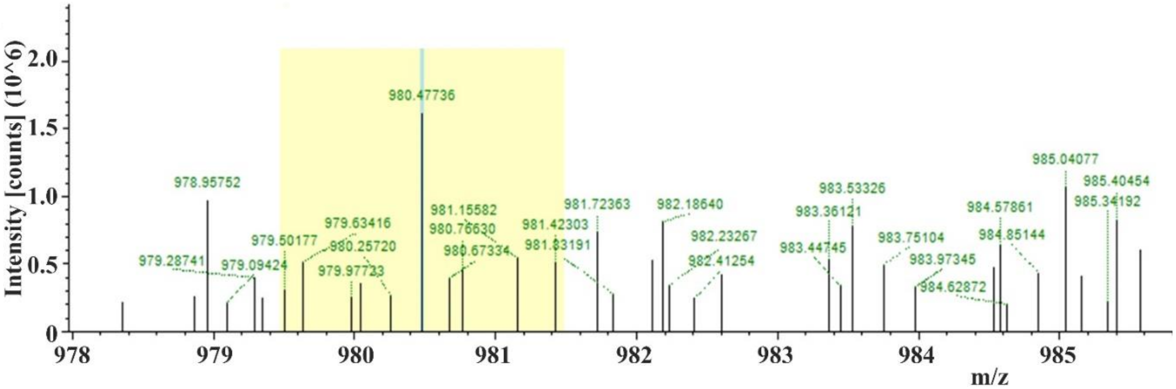

4.

>sp|F4IIUC4|ATCA5_ARATII Alpha carbonic anhydrase 5 OS=Arabidopsis thaliana OX=3702 GN=ACA5 PE=3 SV=1

MKIPSIGYVFFLIFISITIVSSSPDHGEVEDETQFNYEKKGEKGPENWGRLKPEWAMCGK
GNMQSPIDLTDKRVLIDHNLGYLRSQYLPSNATIKNRGHDIMMKFEGGNAGLGITINGTE
YKLQQIHWHSPSEHTLNGKRFVLEEHMVHQSKDGRNAVVAFFYKLGKPDYFLLTLERYLK
RITDTHESQEFVEMVHPRTFGFESKHYYRFIGSLTTPPCSENVIWTISKEMRTVTLKQLI
MLRVTVHDQSNSNARPLQRKNERPVALYIPTWHSKLY

Supplementary Figure 4e. Additional evidence for the identification and localization of the QLIMLR peptide determined. Ion fragment matches **(1)**, fragment matches spectrum **(2)**, monoisotopic ions **(3)**, and the data from FASTA file of αCA5 with sequence of the measured peptide colored in red **(4)** are shown.

1.

Fragment Matches

Value Type: Theo. Mass [Da]

Ion Series | Neutral Losses | Precursor Ions

#1	b^+	Seq.	y^+	#2
1	129.06585	Q		6
2	242.14992	L	645.41163	5
3	355.23398	I	532.32756	4
4	486.27447	M	419.24350	3
5	599.35853	L	288.20302	2
6		R	175.11895	1

2.

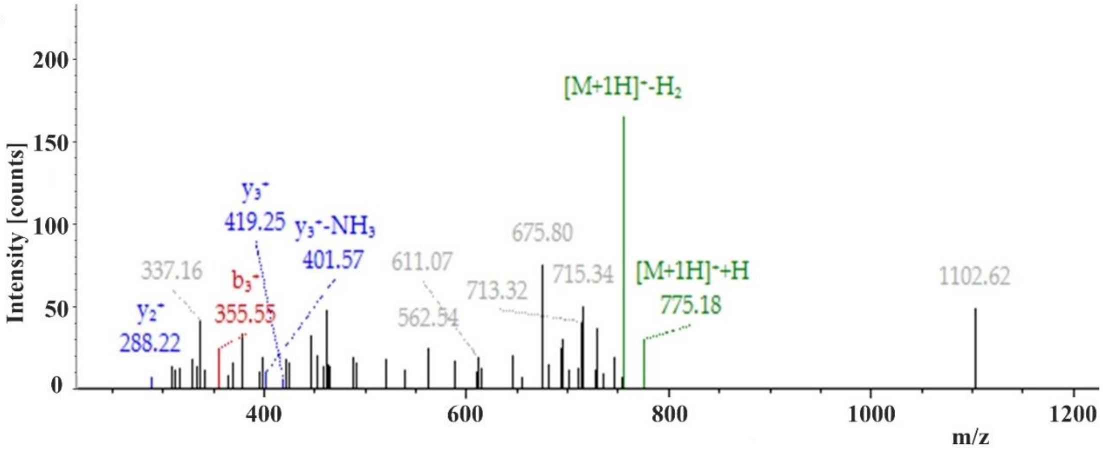

3.

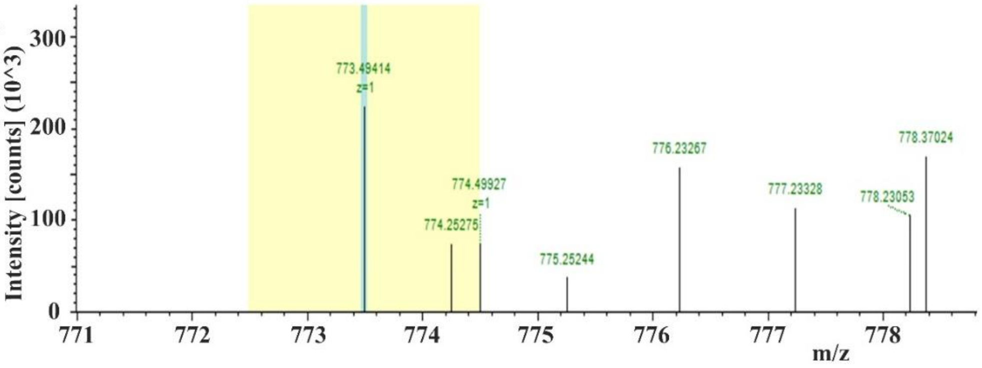

4.

>sp|F4HUC4|ATCA5_ARATH Alpha carbonic anhydrase 5 OS=Arabidopsis thaliana OX=3702 GN=ACA5 PE=3 SV=1

MKIPSIGYVFFLIFISITIVSSSPDHGEVEDETQFNYEKKGEKGPENWGRLKPEWAMCGK
GNMQSPIDLTDKRVLIDHNLGYLRSQYLPSNATIKNRGHDIMMKFEGGNAGLGITINGTE
YKLQQIHWHSPSEHTLNGKRFVLEEHMVHQSKDGRNAVVAFFYKLGKPDYFLLTLERYLK
RITDTHESQEFVEMVHPRTFGFESKHYYRFIGSLTTPPCSENVIWTISKEMRTVTLKQLIMLR
VTVHDQSNSNARPLQRKNERPVALYIPTWHSKLY

Supplementary Table 1. Effect of mafenide on electron transfer rates in thylakoids from two mutant lines (“9-2” and “9-14”, respectively) with knockout gene, encoding αCA5. The basal transfer rate was measured in the presence of 20 µM MV as an acceptor; the coupled transfer rate was measured in the presence of 20 µM MV with the addition of 0.2 mM ADP and 2 mM NaH_2PO_4; the uncoupled transfer rate was measured in the presence of 20 µM MV with the addition of 1 µM gramicidin D.

Electron transfer type	The electron transfer rate, µmol O_2/mg Chl × h			
	9-2 line		9-14 line	
	-	0.1 mM Mafenide	-	0.1 mM Mafenide
Basal	33.3 ± 1.6^{A}	32.9 ± 2.1^{A}	22.6 ± 3.2^{B}	21.8 ± 1.3^{B}
Coupled	77.0 ± 3.8^{A}	74.8 ± 8.2^{A}	41.1 ± 4.0^{B}	40.4 ± 3.1^{B}
Uncoupled	188.4 ± 10.7^{A}	187.4 ± 3.2^{A}	73.6 ± 12.6^{B}	70.9 ± 3.7^{B}

n.d.- not determined.

Data are given as mean values ± SD (n = 6). Similar results were obtained with thylakoids isolated from plants of two independent plantings. Student’s t test (with adjustment by Holm–Bonferroni method) is used for multiple comparisons among four groups (9-2 and 9-14 lines without mafenide and in the presence of 0.1 mM mafenide) for all electron transfer types. Values not connected by the same letter are significantly different (Student’s t test, $P < 0.001$).
